# THE CONCISE GUIDE TO PHARMACOLOGY 2017/18: Ligand‐gated ion channels

**DOI:** 10.1111/bph.13879

**Published:** 2017-10-21

**Authors:** Stephen PH Alexander, John A Peters, Eamonn Kelly, Neil V Marrion, Elena Faccenda, Simon D Harding, Adam J Pawson, Joanna L Sharman, Christopher Southan, Jamie A Davies, CGTP Collaborators

**Affiliations:** ^1^ School of Life Sciences University of Nottingham Medical School Nottingham NG7 2UH UK; ^2^ Neuroscience Division, Medical Education Institute Ninewells Hospital and Medical School University of Dundee Dundee, DD1 9SY UK; ^3^ School of Physiology, Pharmacology and Neuroscience University of Bristol Bristol BS8 1TD UK; ^4^ Centre for Integrative Physiology University of Edinburgh Edinburgh EH8 9XD UK

## Abstract

The Concise Guide to PHARMACOLOGY 2017/18 provides concise overviews of the key properties of nearly 1800 human drug targets with an emphasis on selective pharmacology (where available), plus links to an open access knowledgebase of drug targets and their ligands (www.guidetopharmacology.org), which provides more detailed views of target and ligand properties. Although the Concise Guide represents approximately 400 pages, the material presented is substantially reduced compared to information and links presented on the website. It provides a permanent, citable, point‐in‐time record that will survive database updates. The full contents of this section can be found at http://onlinelibrary.wiley.com/doi/10.1111/bph.13879/full. Ligand‐gated ion channels are one of the eight major pharmacological targets into which the Guide is divided, with the others being: G protein‐coupled receptors, voltage‐gated ion channels, other ion channels, nuclear hormone receptors, catalytic receptors, enzymes and transporters. These are presented with nomenclature guidance and summary information on the best available pharmacological tools, alongside key references and suggestions for further reading. The landscape format of the Concise Guide is designed to facilitate comparison of related targets from material contemporary to mid‐2017, and supersedes data presented in the 2015/16 and 2013/14 Concise Guides and previous Guides to Receptors and Channels. It is produced in close conjunction with the Nomenclature Committee of the Union of Basic and Clinical Pharmacology (NC‐IUPHAR), therefore, providing official IUPHAR classification and nomenclature for human drug targets, where appropriate.

## Conflict of interest

The authors state that there are no conflicts of interest to declare.

## Overview

Ligand‐gated ion channels (LGICs) are integral membrane proteins that contain a pore which allows the regulated flow of selected ions across the plasma membrane. Ion flux is passive and driven by the electrochemical gradient for the permeant ions. These channels are open, or gated, by the binding of a neurotransmitter to an orthosteric site(s) that triggers a conformational change that results in the conducting state. Modulation of gating can occur by the binding of endogenous, or exogenous, modulators to allosteric sites. LGICs mediate fast synaptic transmission, on a millisecond time scale, in the nervous system and at the somatic neuromuscular junction. Such transmission involves the release of a neurotransmitter from a pre‐synaptic neurone and the subsequent activation of post‐synaptically located receptors that mediate a rapid, phasic, electrical signal (the excitatory, or inhibitory, post‐synaptic potential). However, in addition to their traditional role in phasic neurotransmission, it is now established that some LGICs mediate a tonic form of neuronal regulation that results from the activation of extra‐synaptic receptors by ambient levels of neurotransmitter. The expression of some LGICs by non‐excitable cells is suggestive of additional functions.

By convention, the LGICs comprise the excitatory, cation‐selective, nicotinic acetylcholine [54, 257], 5‐HT3 [21, 386], ionotropic glutamate [231, 365] and P2X receptors [174, 349] and the inhibitory, anion‐selective, GABAA [27, 287] and glycine receptors [233, 399]. The nicotinic acetylcholine, 5‐HT3, GABAA and glycine receptors (and an additional zinc‐activated channel) are pentameric structures and are frequently referred to as the Cys‐loop receptors due to the presence of a defining loop of residues formed by a disulphide bond in the extracellular domain of their constituent subunits [259, 353]. However, the prokaryotic ancestors of these receptors contain no such loop and the term pentameric ligand‐gated ion channel (pLGIC) is gaining acceptance in the literature [145]. The ionotropic glutamate and P2X receptors are tetrameric and trimeric structures, respectively. Multiple genes encode the subunits of LGICs and the majority of these receptors are heteromultimers. Such combinational diversity results, within each class of LGIC, in a wide range of receptors with differing pharmacological and biophysical properties and varying patterns of expression within the nervous system and other tissues. The LGICs thus present attractive targets for new therapeutic agents with improved discrimination between receptor isoforms and a reduced propensity for off‐target effects. The development of novel, faster screening techniques for compounds acting on LGICs [100] will greatly aid in the development of such agents.

## Family structure

S131 5‐HT_3_ receptors

S133 Acid‐sensing (proton‐gated) ion channels (ASICs)

S135 Epithelial sodium channels (ENaC)

S137 GABA_A_ receptors

S142 Glycine receptors

S144 Ionotropic glutamate receptors

S149 IP_3_ receptor

S150 Nicotinic acetylcholine receptors

S154 P2X receptors

S156 ZAC

## 5‐HT_3_ receptors

### Overview

The 5‐HT_3_ receptor (nomenclature as agreed by the NC‐IUPHARSubcommittee on 5‐Hydroxytryptamine (serotonin) receptors [157]) is a ligand‐gated ion channel of the Cys‐loop family that includes the zinc‐activated channels, nicotinic acetylcholine, GABA_A_and strychnine‐sensitive glycine receptors. The receptor exists as a pentamer of 4TM subunits that form an intrinsic cation selective channel [21]. Five human 5‐HT_3_ receptor subunits have been cloned and homo‐oligomeric assemblies of 5‐HT_3_A and hetero‐oligomeric assemblies of 5‐HT_3_A and 5‐HT_3_B subunits have been characterised in detail. The 5‐HT_3_C (HTR3C, Q8WXA8), 5‐HT_3_D (HTR3D, Q70Z44) and 5‐HT_3_E (HTR3E, A5X5Y0) subunits [189, 277], like the 5‐HT_3_B subunit, do not form functional homomers, but are reported to assemble with the 5‐HT_3_A subunit to influence its functional expression rather than pharmacological profile [148, 279, 379]. 5‐HT_3_A, ‐C, ‐D, and ‐E subunits also interact with the chaperone RIC‐3 which predominantly enhances the surface expression of homomeric 5‐HT_3_A receptor [379]. The co‐expression of 5‐HT_3_A and 5‐HT_3_C‐E subunits has been demonstrated in human colon [186]. A recombinant hetero‐oligomeric 5‐HT_3_AB receptor has been reported to contain two copies of the 5‐HT_3_A subunit and three copies of the 5‐HT_3_B subunit in the order B‐B‐A‐B‐A [25], but this is inconsistent with recent reports which show at least one A‐A interface [225, 357]. The 5‐HT_3_B subunit imparts distinctive biophysical properties upon hetero‐oligomeric 5‐HT_3_AB versus homo‐oligomeric 5‐HT_3_A recombinant receptors [77, 98, 135, 176, 194, 301, 344], influences the potency of channel blockers, but generally has only a modest effect upon the apparent affinity of agonists, or the affinity of antagonists ([41], but see [76, 81, 98]) which may be explained by the orthosteric binding site residing at an interface formed between 5‐HT_3_A subunits [225, 357]. However, 5‐HT_3_A and 5‐HT_3_AB receptors differ in their allosteric regulation by some general anaesthetic agents, small alcohols and indoles [158, 317, 341]. The potential diversity of 5‐HT_3_ receptors is increased by alternative splicing of the genes HTR_3_A and E [44, 151, 276, 278, 279]. In addition, the use of tissue‐specific promoters driving expression from different transcriptional start sites has been reported for the HTR3A, HTR3B, HTR3D and HTR3E genes, which could result in 5‐HT_3_ subunits harbouring different N‐termini [176, 276, 366]. To date, inclusion of the 5‐HT_3_A subunit appears imperative for 5‐HT_3_ receptor function.

**Table:** 

Nomenclature	5‐HT_3_AB	5‐HT_3_A
Subunits	5‐HT_3_A, 5‐HT_3_B	5‐HT_3_A
Selective agonists	–	meta‐chlorphenylbiguanide [26, 77, 214, 262, 263], 2‐methyl‐5‐HT [26, 77, 214, 262], SR57227A [102] – Rat, 1‐phenylbiguanide [26]
Antagonists	–	vortioxetine (p*K*_i_ 8.4) [18], metoclopramide (p*K*_i_ 6–6.4) [41, 152]
Selective antagonists	–	palonosetron (p*K*_i_ 10.5) [269], alosetron (p*K*_i_ 9.5) [146], (S)‐zacopride (p*K*_i_ 9) [41], granisetron (p*K*_i_∼8.6–8.8) [152, 262], tropisetron (p*K*_i_ 8.5–8.8) [214, 262], ondansetron (p*K*_i_∼7.8–8.3) [41, 152, 262]
Channel blockers	picrotoxinin (pIC_50_ 4.2) [352], bilobalide (pIC_50_ 2.5) [352], ginkgolide B (pIC_50_ 2.4) [352]	picrotoxinin (pIC_50_ 5) [351], TMB‐8 (pIC_50_ 4.9) [348], diltiazem (pIC_50_ 4.7) [351], bilobalide (pIC_50_ 3.3) [351], ginkgolide B (pIC_50_ 3.1) [351]
Labelled ligands	–	[^3^H]ramosetron (Antagonist) (p*K*_d_ 9.8) [262], [^3^H]GR65630 (Antagonist) (p*K*_d_ 8.6–9.3) [146, 214], [^3^H]granisetron (Antagonist) (p*K*_d_ 8.9) [41, 152], [^3^H](S)‐zacopride (Antagonist) (p*K*_d_ 8.7) [293], [^3^H]LY278584 (Antagonist) (p*K*_d_ 8.5) [1]
Functional Characteristics	*γ* = 0.4‐0.8 pS [+ 5‐HT3B, *γ* = 16 pS]; inwardly rectifying current [+ 5‐HT3B, rectification reduced]; n_H_ 2‐3 [+ 5‐HT3B 1‐2]; relative permeability to divalent cations reduced by co‐expression of the 5‐HT3B subunit	*γ* = 0.4‐0.8 pS [+ 5‐HT3B, *γ* = 16 pS]; inwardly rectifying current [+ 5‐HT3B, rectification reduced]; n_H_ 2‐3 [+ 5‐HT3B 1‐2]; relative permeability to divalent cations reduced by co‐expression of the 5‐HT3B subunit

## Subunits

**Table:** 

Nomenclature	5‐HT3A	5‐HT3B	5‐HT3C	5‐HT3D	5‐HT3E
HGNC, UniProt	HTR3A, P46098	HTR3B, O95264	HTR3C, Q8WXA8	HTR3D, Q70Z44	HTR3E, A5X5Y0
Functional Characteristics	*γ* = 0.4‐0.8 pS [+ 5‐HT3B, *γ* = 16 pS]; inwardly rectifying current [+ 5‐HT3B, rectification reduced]; n_H_ 2‐3 [+ 5‐HT3B 1‐2]; relative permeability to divalent cations reduced by co‐expression of the 5‐HT3B subunit	*γ* = 0.4‐0.8 pS [+ 5‐HT3B, *γ* = 16 pS]; inwardly rectifying current [+ 5‐HT3B, rectification reduced]; n_H_ 2‐3 [+ 5‐HT3B 1‐2]; relative permeability to divalent cations reduced by co‐expression of the 5‐HT3B subunit	–	–	–

### Comments

Quantitative data in the table refer to homo‐oligomeric assemblies of the human 5‐HT_3_A subunit, or the receptor native to human tissues. Significant changes introduced by co‐expression of the 5‐HT_3_B subunit are indicated in parenthesis. Although not a selective antagonist, methadone displays multimodal and subunit‐dependent antagonism of 5‐HT_3_receptors [81]. Similarly, TMB‐8, diltiazem, picrotoxin, bilobalide and ginkgolide B are not selective for 5‐HT_3_ receptors (e.g.[352]). The anti‐malarial drugs mefloquine and quinine exert a modestly more potent block of 5‐HT_3_A versus 5‐HT_3_AB receptor‐mediated responses [354]. Known better as a partial agonist of nicotinic acetylcholine *α*4*β*2 receptors, varenicline is also an agonist of the 5‐HT_3_A receptor [231]. Human [26, 262], rat [164], mouse [243], guinea‐pig [214] ferret [264] and canine [178] orthologues of the 5‐HT_3_A receptor subunit have been cloned that exhibit intraspecies variations in receptor pharmacology. Notably, most ligands display significantly reduced affinities at the guinea‐pig 5‐HT_3_ receptor in comparison with other species. In addition to the agents listed in the table, native and recombinant 5‐HT_3_ receptors are subject to allosteric modulation by extracellular divalent cations, alcohols, several general anaesthetics and 5‐hydroxy‐ and halide‐substituted indoles (see reviews [294, 355, 356, 380]).

### Further reading on 5‐HT_3_ receptors

Andrews, PL et al. (2014) Nausea and the quest for the perfect anti‐emetic. Eur J Pharmacol 722: 108‐21 [PMID:24157981]

Fakhfouri, G et al. (2015) From Chemotherapy‐Induced Emesis to Neuroprotection: Therapeutic Opportunities for 5‐HT3 Receptor Antagonists. Mol Neurobiol 52: 1670‐1679 [PMID:25377794]

Gupta, D et al. (2016) 5HT3 receptors: Target for new antidepressant drugs. Neurosci Biobehav Rev 64: 311‐25 [PMID:26976353]

Hoyer, D et al. (1994) International Union of Pharmacology classification of receptors for 5‐hydroxytryptamine (Serotonin). Pharmacol Rev 46: 157‐203

Lochner, M et al. (2015) A review of fluorescent ligands for studying 5‐HT3 receptors. Neuropharmacology 98: 31‐40 [PMID:25892507]

Rojas, C et al. (2014) Molecular mechanisms of 5‐HT(3) and NK(1) receptor antagonists in prevention of emesis. Eur J Pharmacol 722: 26‐37 [PMID:24184669]

## Acid‐sensing (proton‐gated) ion channels (ASICs)

### Overview

Acid‐sensing ion channels (ASICs, **nomenclature as agreed by NC‐IUPHAR**[193]) are members of a Na^+^ channel superfamily that includes the epithelial Na^+^ channel (ENaC), the FMRF‐amide activated channel (FaNaC) of invertebrates, the degenerins (DEG) of Caenorhabitis elegans, channels in Drosophila melanogaster and 'orphan' channels that include BLINaC 325 and INaC [323] that have also been named BASICs, for bile acid‐activated ion channels [383]. ASIC subunits contain two TM domains and assemble as homo‐ or hetero‐trimers [17, 125, 175] to form proton‐gated, voltage‐insensitive, Na^+^ permeable, channels (reviewed in [131, 382]). Splice variants of ASIC1 [provisionally termed ASIC1a (ASIC, ASIC*α*, BNaC2*α*) [376], ASIC1b (ASIC*β*, BNaC2*β*) [61] and ASIC1b2 (ASIC*β*2) [367]; note that ASIC1a is also permeable to Ca^2+^] and ASIC2 [provisionally termed ASIC2a (MDEG1, BNaC1*α*, BNC1*α*) [121, 308, 377] and ASIC2b (MDEG2, BNaC1*β*) [223]] have been cloned. Unlike ASIC2a (listed in table), heterologous expression of ASIC2b alone does not support H^+^‐gated currents. A third member, ASIC3 (DRASIC, TNaC1) [375], has been identified. A fourth mammalian member of the family (ASIC4/SPASIC) does not support a proton‐gated channel in heterologous expression systems and is reported to downregulate the expression of ASIC1a and ASIC3 [1, 92, 130, 222]. ASIC channels are primarily expressed in central and peripheral neurons including nociceptors where they participate in neuronal sensitivity to acidosis. They have also been detected in taste receptor cells (ASIC1‐3), photoreceptors and retinal cells (ASIC1‐3), cochlear hair cells (ASIC1b), testis (hASIC3), pituitary gland (ASIC4), lung epithelial cells (ASIC1a and ‐3), urothelial cells, adipose cells (ASIC3), vascular smooth muscle cells (ASIC1‐3), immune cells (ASIC1,‐3 and ‐4) and bone (ASIC1‐3). A neurotransmitter‐like function of protons has been suggested, involving postsynaptically located ASICs of the CNS in functions such as learning and fear perception [97, 207, 408], responses to focal ischemia [390] and autoimmune inflammation [115], as well as seizures [408] and pain [37, 84, 85, 89]. Heterologously expressed heteromultimers form ion channels with differences in kinetics, ion selectivity, pH‐ sensitivity and sensitivity to blockers that resemble some of the native proton activated currents recorded from neurones [15, 24, 107, 223].

**Table:** 

Nomenclature	ASIC1	ASIC2
HGNC, UniProt	ASIC1, P78348	ASIC2, Q16515
Endogenous activators	Extracellular H^+^ (ASIC1a) (pEC_50_∼6.2–6.8), Extracellular H^+^ (ASIC1b) (pEC_50_∼5.1–6.2)	Extracellular H^+^ (pEC_50_∼4.1–5)
Channel blockers	psalmotoxin 1 (ASIC1a) (pIC_50_ 9), Zn^2+^ (ASIC1a) (pIC_50_∼8.2), Pb^2+^ (ASIC1b) (pIC_50_∼5.8), A‐317567 (ASIC1a) (pIC_50_∼5.7) [99] – Rat, Pb^2+^ (ASIC1a) (pIC_50_∼5.4), amiloride (ASIC1a) (pIC_50_ 5), benzamil (ASIC1a) (pIC_50_ 5), ethylisopropylamiloride (ASIC1a) (pIC_50_ 5), nafamostat (ASIC1a) (pIC_50_∼4.9), amiloride (ASIC1b) (pIC_50_ 4.6–4.7), flurbiprofen (ASIC1a) (pIC_50_ 3.5) [372] – Rat, ibuprofen (ASIC1a) (pIC_50_∼3.5), Ni^2+^ (ASIC1a) (pIC_50_∼3.2)	amiloride (pIC_50_ 4.6), A‐317567 (pIC_50_∼4.5), nafamostat (pIC_50_∼4.2), Cd^2+^ (pIC_50_∼3)
Labelled ligands	[^125^I]psalmotoxin 1 (ASIC1a) (p*K*_d_ 9.7)	–
Functional Characteristics	ASIC1a: *γ* ^~^ 14pS P_Na_/P_K_ = 5‐13, P_Na_/P_Ca_ =2.5 rapid activation rate (5.8‐13.7 ms), rapid inactivation rate (1.2‐4 s) @ pH 6.0, slow recovery (5.3‐13s) @ pH 7.4 ASIC1b: *γ* ^~^ 19 pS P_Na_/P_K_ =14.0, P_Na_≫ P_Ca_ rapid activation rate (9.9 ms), rapid inactivation rate (0.9‐1.7 s) @ pH 6.0, slow recovery (4.4‐7.7 s) @ pH 7.4	*γ* ^~^ 10.4‐13.4 pS P_Na_/P_K_ =10, P_Na_/P_Ca_ = 20 rapid activation rate, moderate inactivation rate (3.3‐5.5 s) @ pH 5
Comments	ASIC1a and ASIC1b are also blocked by diarylamidines (IC_50_^~^3 *μ*M for ASIC1a)	ASIC2 is also blocked by diarylamidines

**Table:** 

Nomenclature	ASIC3
HGNC, UniProt	ASIC3, Q9UHC3
Endogenous activators	Extracellular H^+^ (transient component) (pEC_50_∼6.2–6.7), Extracellular H^+^ (sustained component) (pEC_50_∼3.5–4.3)
Activators	GMQ (largly non‐desensitizing; at pH 7.4) (pEC_50_∼3), arcaine (at pH 7.4) (pEC_50_∼2.9), agmatine (at pH 7.4) (pEC_50_∼2)
Channel blockers	APETx2 (transient component only) (pIC_50_ 7.2), nafamostat (transient component) (pIC_50_∼5.6), A‐317567 (pIC_50_∼5), amiloride (transient component only ‐ sustained component enhanced by 200*μ*M amiloride at pH 4) (pIC_50_ 4.2–4.8), Gd^3+^ (pIC_50_ 4.4), Zn^2+^ (pIC_50_ 4.2), aspirin (sustained component) (pIC_50_ 4) [372], diclofenac (sustained component) (pIC_50_ 4), salicylic acid (sustained component) (pIC_50_ 3.6)
Functional Characteristics	*γ* ^~^ 13‐15 pS; biphasic response consisting of rapidly inactivating transient and sustained components; very rapid activation (<5 ms) and inactivation (0.4 s); fast recovery (0.4‐0.6 s) @ pH 7.4, transient component partially inactivated at pH 7.2
Comments	ASIC3 is also blocked by diarylamidines

### Comments

psalmotoxin 1 (PcTx1) inhibits ASIC1a by increasing the affinity to H+ and promoting channel desensitization [64, 107]. PcTx1 has little effect on ASIC2a, ASIC3 or ASIC1a expressed as a heteromultimer with either ASIC2a, or ASIC3 but does inhibit ASIC1a expressed as a heteromultimer with ASIC2b [330]. ASIC1‐containing homo‐ and heteromers are inhibited by Mambalgins, toxins contained in the black mamba venom, which induce in ASIC1a an acidic shift of the pH dependence of activation [89]. APETx2 most potently blocks homomeric ASIC3 channels, but also ASIC2b+ASIC3, ASIC1b+ASIC3, and ASIC1a+ASIC3 heteromeric channels with IC_50_ values of 117 nM, 900 nM and 2 μM, respectively. APETx2 has no effect on ASIC1a, ASIC1b, ASIC2a, or ASIC2a+ASIC3 [88, 90]. APETx2 inhibits however also voltage‐gated Na+ channels [34, 297]. IC_50_ values for A‐317567 are inferred from blockade of ASIC channels native to dorsal root ganglion neurones [99]. The pEC_50_ values for proton activation of ASIC channels are influenced by numerous factors including extracellular di‐ and poly‐valent ions, Zn^2+^, protein kinase C and serine proteases (reviewed in [193, 382]). Rapid acidification is required for activation of ASIC1 and ASIC3 due to fast inactivation/desensitization. pEC_50_values for H^+^‐activation of either transient, or sustained, currents mediated by ASIC3 vary in the literature and may reflect species and/or methodological differences [16, 79, 375]. The transient ASIC current component is Na^+^‐selective (PNa/PK of about 10) [375, 392] whereas the sustained current component that is observed with ASIC3 and some ASIC heteromers is non‐selective between Na^+^ and K^+^[79]. The reducing agents dithiothreitol (DTT) and glutathione (GSH) increase ASIC1a currents expressed in CHO cells and ASIC‐like currents in sensory ganglia and central neurons [8, 68] whereas oxidation, through the formation of intersubunit disulphide bonds, reduces currents mediated by ASIC1a [405]. ASIC1a is also irreversibly modulated by extracellular serine proteases, such as trypsin, through proteolytic cleavage [373]. Non‐steroidal anti‐inflammatory drugs (NSAIDs) are direct inhibitors of ASIC currents (reviewed in [22]). Extracellular Zn^2+^ potentiates proton activation of homomeric and heteromeric channels incorporating ASIC2a, but not homomeric ASIC1a or ASIC3 channels [23]. However, removal of contaminating Zn^2+^ by chelation reveals a high affinity block of homomeric ASIC1a and heteromeric ASIC1a+ASIC2 channels by Zn^2+^ indicating complex biphasic actions of the divalent [69]. Nitric oxide potentiates submaximal currents activated by H^+^ mediated by ASIC1a, ASIC1b, ASIC2a and ASIC3 [47]. Ammonium ions activate ASIC channels (most likely ASIC1a) in midbrain dopaminergic neurones: that may be relevant to neuronal disorders associated with hyperammonemia [302]. The positive modulation of homomeric, heteromeric and native ASIC channels by the peptide FMRFamide and related substances, such as neuropeptides FF and SF, is reviewed in detail in [369]. Inflammatory conditions and particular pro‐inflammatory mediators such as arachidonic acid induce overexpression of ASIC‐encoding genes and enhance ASIC currents [85, 241, 337]. The sustained current component mediated by ASIC3 is potentiated by hypertonic solutions in a manner that is synergistic with the effect of arachidonic acid [85]. ASIC3 is partially activated by the lipids lysophosphatidylcholine (LPC) and arachidonic acid [244]. Mit‐Toxin, which is contained in the venom of the Texas coral snake, activates several ASIC subtypes [37]. Selective activation of ASIC3 by GMQ at a site separate from the proton binding site is potentiated by mild acidosis and reduced extracellular Ca^2+^[402].

### Further reading on Acid‐sensing (proton‐gated) ion channels (ASICs)

Baron A et al. (2015) Pharmacology of acid‐sensing ion channels ‐ Physiological and therapeutical perspectives. Neuropharmacology 94: 19‐35 [PMID:25613302]

Boscardin E et al. (2016) The function and regulation of acid‐sensing ion channels (ASICs) and the epithelial Na(+) channel (ENaC): IUPHAR Review 19. Br J Pharmacol 173: 2671‐701 [PMID:27278329]

Grunder S et al. (2015) Biophysical properties of acid‐sensing ion channels (ASICs). Neuropharmacology 94: 9‐18 [PMID:25585135]

Kellenberger S et al. (2015) International Union of Basic and Clinical Pharmacology. XCI. structure, function, and pharmacology of acid‐sensing ion channels and the epithelial Na+ channel. Pharmacol Rev 67: 1‐35 [PMID:25287517]

Osmakov DI et al. (2014) Acid‐sensing ion channels and their modulators. Biochemistry (Mosc) 79: 1528‐45 [PMID:25749163]

## Epithelial sodium channels (ENaC)

### Overview

The epithelial sodium channels (ENaC) mediate sodium reabsorption in the aldosterone‐sensitive distal part of the nephron and the collecting duct of the kidney. ENaC is assembled as a heterotrimer composed of three subunits *α*, *β*, and *γ* or *δ*, *β*, and *γ*[137]. These subunits consitute a family within the ENaC/Degenerin super‐family [137]. Genes encoding ENaC subunits are found in all vertebrates with the exception of ray‐finned fishes [137]. ENaC composed of *α*, *β*, and *γ* subunits is located mostly in tight or high‐resistance epithelial tissues such as the airways, distal colon and exocrine glands [104]. ENaC activity is tightly regulated in the kidney by aldosterone, angiotensin II(AGT, P01019), vasopressin(AVP, P01185), insulin(INS, P01308) and glucocorticoids; this fine regulation of ENaC is essential to maintain sodium balance between daily intake and urinary excretion of sodium, circulating volume and blood pressure. ENaC expression is also vital for clearance of foetal lung fluid, and to maintain air‐surface‐liquid [160, 227]. Sodium reabsorption is suppressed by the ‘potassium‐sparing’ diuretics amiloride and triamterene. ENaC is a heteromultimeric channel made of homologous *α* *β* and *γ* subunits. The primary structure of the *α* ENaC subunit was identified by expression cloning [48, 137]; *β* and *γ* ENaC subunits were identified by functional complementation of the *α* subunit [49, 137]. Each ENaC subunit contains 2 TM *α* helices connected by a large extracellular loop and short cytoplasmic amino‐ and carboxy‐termini. The stoichiometry of the epithelial sodium channel in the kidney and related epithelia is, by homology with the structurally related channel ASIC1a, thought to be a heterotrimer of 1*α*:1*β*:1*γ* subunits [125].

**Table:** 

Nomenclature	ENaC*αβγ*
Subunits	ENaC *β*, ENaC *α*, ENaC *γ*
Activators	S3969 (pEC_50_ 5.9) [229]
Channel blockers	P552‐02 (pIC_50_ 8.1), benzamil (pIC_50_∼8), amiloride (pIC_50_ 6.7–7), triamterene (pIC_50_∼5.3) [49, 192]
Functional Characteristics	*γ*≈4‐5 pS, P_Na_/P_K_> 20; tonically open at rest; expression and ion flux regulated by circulating aldosterone‐mediated changes in gene transcription. The action of aldosterone, which occurs in ‘early’ (1.5–3 h) and ‘late’ (6‐24 hr) phases is competitively antagonised by spironolactone, its active metabolites and eplerenone. Glucocorticoids are important functional regulators in lung/airways and this control is potentiated by thyroid hormone; but the mechanism underlying such potentiation is unclear [19, 311, 322]. The density of channels in the apical membrane, and hence G_Na_, can be controlledvia both serum and glucocorticoid‐regulated kinases (SGK1, 2 and 3) [80, 114] and via cAMP/PKA [267]; and these protein kinases appear to act by inactivating Nedd‐4/2, a ubiquitin ligase that normally targets the ENaC channel complex for internalization and degradation [35, 80]. ENaC is constitutively activated by soluble and membrane‐bound serine proteases, such as furin, prostasin (CAP1), plasmin and elastase [202, 203, 305, 313, 314]. The activation of ENaC by proteases is blocked by a protein, SPLUNC1, secreted by the airways and which binds specifically to ENaC to prevent its cleavage [120]. Pharmacological inhibitors of proteases (e.g. camostat acting upon prostasin) reduce the activity of ENaC [237]. Phosphatidylinositides such as PtIns(4,5)P_2_ and PtIns(3,4,5)P_3_) stabilise channel gating probably by binding to the *β* and *γ* ENaC subunits, respectively [235, 307], whilst C terminal phosphorylation of *β* and *γ*‐ENaC by ERK1/2 has been reported to inhibit the withdrawal of the channel complex from the apical membrane [393]. This effect may contribute to the cAMP‐mediated increase in sodium conductance.

## Subunits

**Table:** 

Nomenclature	ENaC *α*	ENaC *β*	ENaC *δ*	ENaC *γ*
HGNC, UniProt	SCNN1A, P37088	SCNN1B, P51168	SCNN1D, P51172	SCNN1G, P51170

### Comments

Data in the table refer to the *α* *β* *γ* heteromer. There are several human diseases resulting from mutations in ENaC subunits [137]. Liddle's syndrome (including features of salt‐sensitive hypertension and hypokalemia), is associated with gain of function mutations in the *β* and *γ* subunits leading to defective ENaC ubiquitylation and increased stability of active ENaC at the cell surface [137, 314, 324, 343]. Enzymes that deubiquitylate ENaC increase its function in vivo. Pseudohypoaldosteronism type 1 (PHA‐1) can occur through either mutations in the gene encoding the mineralocorticoid receptor, or loss of function mutations in genes encoding ENaC subunits [39, 137]. Regulation of ENaC by phosphoinositides may underlie insulin(INS, P01308)‐evoked renal Na^+^ retention that can complicate the clinical management of type 2 diabetes using insulin‐sensitizing thiazolidinedione drugs [132].

### Further reading on Epithelial sodium channels (ENaC)

Boscardin E et al. (2016) The function and regulation of acid‐sensing ion channels (ASICs) and the epithelial Na(+) channel (ENaC): IUPHAR Review 19. Br J Pharmacol 173: 2671‐701 [PMID:27278329]

Eaton DC et al. (2009) The contribution of epithelial sodium channels to alveolar function in health and disease. Annu Rev Physiol 71: 403‐23 [PMID:18831683]

Kellenberger S et al. (2015) International Union of Basic and Clinical Pharmacology. XCI. structure, function, and pharmacology of acid‐sensing ion channels and the epithelial Na+ channel. Pharmacol Rev 67: 1‐35 [PMID:25287517]

Kleyman TR et al. (2009) ENaC at the cutting edge: regulation of epithelial sodium channels by proteases. J Biol Chem 284: 20447‐51 [PMID:19401469]

Soundararajan, R et al. (2010) Role of epithelial sodium channels and their regulators in hypertension . J Biol Chem 285: 30363‐9 [PMID:20624922]

## GABA_A_ receptors

### Overview

The GABA_A_ receptor is a ligand‐gated ion channel of the Cys‐loop family that includes the nicotinic acetylcholine, 5‐HT_3_ and strychnine‐sensitive glycine receptors. GABA_A_ receptor‐mediated inhibition within the CNS occurs by fast synaptic transmission, sustained tonic inhibition and temporally intermediate events that have been termed ‘GABA_A_, slow’ [51]. GABA_A_ receptors exist as pentamers of 4TM subunits that form an intrinsic anion selective channel. Sequences of six *α*, three *β*, three *γ*, one *δ*, three *ρ*, one *ϵ*, one *π* and one *θ* GABA_A_ receptor subunits have been reported in mammals [286, 287, 331, 333]. The *π*‐subunit is restricted to reproductive tissue. Alternatively spliced versions of many subunits exist (e.g. *α*4‐ and *α*6‐ (both not functional) *α*5‐, *β*2‐, *β*3‐ and *γ*2), along with RNA editing of the *α*3 subunit [75]. The three *ρ*‐subunits, (*ρ*1‐3) function as either homo‐ or hetero‐oligomeric assemblies [60, 406]. Receptors formed from *ρ*‐subunits, because of their distinctive pharmacology that includes insensitivity to bicuculline, benzodiazepines and barbiturates, have sometimes been termed GABA_C_ receptors [406], but they are classified as GABA_A_ receptors by NC‐IUPHAR on the basis of structural and functional criteria [20, 286, 287].

Many GABA_A_ receptor subtypes contain *α*‐, *β*‐ and *γ*‐subunits with the likely stoichiometry 2*α*.2*β*.1*γ*[206, 287]. It is thought that the majority of GABA_A_ receptors harbour a single type of *α*‐ and *β* ‐subunit variant. The *α*1*β*2*γ*2 hetero‐oligomer constitutes the largest population of GABA_A_ receptors in the CNS, followed by the *α*2*β*3*γ*2 and *α*3*β*3*γ*2 isoforms. Receptors that incorporate the *α*4‐ *α*5‐or *α*6‐subunit, or the *β*1‐, *γ*1‐, *γ*3‐, *δ*‐, *ϵ*‐ and *θ*‐subunits, are less numerous, but they may nonetheless serve important functions. For example, extrasynaptically located receptors that contain *α*6‐ and *δ*‐subunits in cerebellar granule cells, or an *α*4‐ and *δ*‐subunit in dentate gyrus granule cells and thalamic neurones, mediate a tonic current that is important for neuronal excitability in response to ambient concentrations of GABA [27, 109, 265, 327, 338]. GABA binding occurs at the *β*+/*α*‐ subunit interface and the homologous *γ*+/*α*‐ subunits interface creates the benzodiazepine site. A second site for benzodiazepine binding has recently been postulated to occur at the *α*+/*β*‐ interface ([310]; reviewed by [332]). The particular *α*‐and *γ*‐subunit isoforms exhibit marked effects on recognition and/or efficacy at the benzodiazepine site. Thus, receptors incorporating either *α*4‐ or *α*6‐subunits are not recognised by ‘classical’ benzodiazepines, such as flunitrazepam(but see [400]). The trafficking, cell surface expression, internalisation and function of GABA_A_ receptors and their subunits are discussed in detail in several recent reviews [66, 166, 232, 371] but one point worthy of note is that receptors incorporating the *γ*2 subunit (except when associated with *α*5) cluster at the postsynaptic membrane (but may distribute dynamically between synaptic and extrasynaptic locations), whereas as those incorporating the d subunit appear to be exclusively extrasynaptic.

NC‐IUPHAR [20, 287] class the GABA_A_ receptors according to their subunit structure, pharmacology and receptor function. Currently, eleven native GABA_A_ receptors are classed as conclusively identified (i.e., *α*1*β*2*γ*2, *α*1*β* *γ*2, *α*3*β* *γ*2, *α*4*β* *γ*2, *α*4*β*2*δ*, *α*4*β*3*δ*, *α*5*β* *γ*2, *α*6*β* *γ*2, *α*6*β*2*δ*, *α*6*β*3*δ* and *ρ*) with further receptor isoforms occurring with high probability, or only tentatively [286, 287]. It is beyond the scope of this Guide to discuss the pharmacology of individual GABA_A_ receptor isoforms in detail; such information can be gleaned in the reviews [20, 117, 181, 206, 209, 270, 286, 287, 331] and [11, 12]. Agents that discriminate between *α*‐subunit isoforms are noted in the table and additional agents that demonstrate selectivity between receptor isoforms, for example via *β*‐subunit selectivity, are indicated in the text below. The distinctive agonist and antagonist pharmacology of *ρ* receptors is summarised in the table and additional aspects are reviewed in [60, 182, 274, 406].

**Table:** 

Nomenclature	GABA_A_ receptor *α*1 subunit	GABA_A_ receptor *α*2 subunit
HGNC, UniProt	GABRA1, P14867	GABRA2, P47869
Agonists	gaboxadol [GABA site], isoguvacine [GABA site], isonipecotic acid [GABA site], muscimol [GABA site], piperidine‐4‐sulphonic acid [GABA site]	gaboxadol [GABA site], isoguvacine [GABA site], isonipecotic acid [GABA site], muscimol [GABA site], piperidine‐4‐sulphonic acid [GABA site]
Selective antagonists	bicuculline [GABA site], gabazine [GABA site]	bicuculline [GABA site], gabazine [GABA site]
Channel blockers	TBPS, picrotoxin	TBPS, picrotoxin
Endogenous allosteric modulators	5*α*‐pregnan‐3*α*‐ol‐20‐one (Potentiation), Zn^2+^ (Inhibition), tetrahydrodeoxycorticosterone (Potentiation)	5*α*‐pregnan‐3*α*‐ol‐20‐one (Potentiation), Zn^2+^ (Inhibition), tetrahydrodeoxycorticosterone (Potentiation)
Allosteric modulators	flumazenil [benzodiazepine site] (Antagonist) (p*K*_i_ 9.1) [159], clonazepam (Positive) (p*K*_i_ 8.9) [309], flunitrazepam [benzodiazepine site] (Positive) (p*K*_i_ 8.3) [133], diazepam [benzodiazepine site] (Positive) (p*K*_i_ 7.8) [309], alprazolam [benzodiazepine site] (Positive) (pEC_50_ 7.4) [5], *α*3IA [benzodiazepine site] (Inverse agonist), *α*5IA [benzodiazepine site] (Inverse agonist), DMCM [benzodiazepine site] (Inverse agonist)	flumazenil [benzodiazepine site] (Antagonist) (p*K*_i_ 9.1) [159], clonazepam (Positive) (p*K*_i_ 8.8) [309], flunitrazepam [benzodiazepine site] (Positive) (p*K*_i_ 8.3) [133], alprazolam [benzodiazepine site] (Positive) (pEC_50_ 7.9) [5], diazepam [benzodiazepine site] (Positive) (p*K*_i_ 7.8) [309], *α*3IA [benzodiazepine site] (Inverse agonist), *α*5IA [benzodiazepine site] (Inverse agonist), DMCM [benzodiazepine site] (Inverse agonist)
Selective allosteric modulators	zolpidem (Positive) (p*K*_i_ 7.4–7.7) [134, 325], L838417 [benzodiazepine site] (Antagonist), ZK93426 [benzodiazepine site] (Antagonist), indiplon [benzodiazepine site] (Full agonist), ocinaplon [benzodiazepine site] (Full agonist)	L838417 [benzodiazepine site] (Partial agonist), TPA023 [benzodiazepine site] (Partial agonist)
Labelled ligands	[^11^C]flumazenil [benzodiazepine site] (Allosteric modulator, Antagonist), [^18^F]fluoroethylflumazenil [benzodiazepine site] (Allosteric modulator, Antagonist), [^35^S]TBPS [anion channel] (Channel blocker), [^3^H]CGS8216 [benzodiazepine site] (Allosteric modulator, Mixed), [^3^H]flunitrazepam [benzodiazepine site] (Allosteric modulator, Positive), [^3^H]gabazine [GABA site] (Antagonist), [^3^H]muscimol [GABA site] (Agonist), [^3^H]zolpidem [benzodiazepine site] (Allosteric modulator, Positive)	[^11^C]flumazenil [benzodiazepine site] (Allosteric modulator, Antagonist), [^18^F]fluoroethylflumazenil [benzodiazepine site] (Allosteric modulator, Antagonist), [^35^S]TBPS [anion channel] (Channel blocker), [^3^H]CGS8216 [benzodiazepine site] (Allosteric modulator, Mixed), [^3^H]flunitrazepam [benzodiazepine site] (Allosteric modulator, Full agonist), [^3^H]gabazine [GABA site] (Antagonist), [^3^H]muscimol [GABA site] (Agonist)
Comments	Zn^2+^ is an endogenous allosteric regulator and causes potent inhibition of receptors formed from binary combinations of *α* and *β* subunits, incorporation of a *δ* or *γ* subunit causes a modest, or pronounced, reduction in inhibitory potency, respectively [208]	Zn^2+^ is an endogenous allosteric regulator and causes potent inhibition of receptors formed from binary combinations of *α* and *β* subunits, incorporation of a *δ* or *γ* subunit causes a modest, or pronounced, reduction in inhibitory potency, respectively [208]

**Table:** 

Nomenclature	GABA_A_ receptor *α*3 subunit	GABA_A_ receptor *α*4 subunit
HGNC, UniProt	GABRA3, P34903	GABRA4, P48169
Agonists	gaboxadol [GABA site], isoguvacine [GABA site], isonipecotic acid [GABA site], muscimol [GABA site], piperidine‐4‐sulphonic acid [GABA site]	gaboxadol [GABA site], isoguvacine [GABA site], muscimol [GABA site], piperidine‐4‐sulphonic acid [GABA site] (low efficacy)
Selective agonists	–	isonipecotic acid [GABA site] (relatively high efficacy)
Selective antagonists	bicuculline [GABA site], gabazine [GABA site]	bicuculline [GABA site], gabazine [GABA site]
Channel blockers	TBPS, picrotoxin	TBPS, picrotoxin
Endogenous allosteric modulators	5*α*‐pregnan‐3*α*‐ol‐20‐one (Potentiation), Zn^2+^ (Inhibition), tetrahydrodeoxycorticosterone (Potentiation)	5*α*‐pregnan‐3*α*‐ol‐20‐one (Potentiation), Zn^2+^ (Inhibition), tetrahydrodeoxycorticosterone (Potentiation)
Allosteric modulators	flumazenil [benzodiazepine site] (Antagonist) (p*K*_i_ 9) [159], clonazepam (Positive) (p*K*_i_ 8.7) [309], flunitrazepam [benzodiazepine site] (Positive) (p*K*_i_ 7.8) [133], diazepam [benzodiazepine site] (Positive) (p*K*_i_ 7.8) [309], alprazolam [benzodiazepine site] (Positive) (pEC_50_ 7.2) [5], *α*5IA [benzodiazepine site] (Inverse agonist), DMCM [benzodiazepine site] (Inverse agonist)	–
Selective allosteric modulators	*α*3IA [benzodiazepine site] (higher affinity), L838417 [benzodiazepine site] (Partial agonist), Ro19‐4603 [benzodiazepine site] (Inverse agonist), TP003 [benzodiazepine site] (Partial agonist), TPA023 [benzodiazepine site] (Partial agonist)	Ro15‐4513 [benzodiazepine site] (Full agonist), bretazenil [benzodiazepine site] (Full agonist)
Labelled ligands	[^11^C]flumazenil [benzodiazepine site] (Allosteric modulator, Antagonist), [^18^F]fluoroethylflumazenil [benzodiazepine site] (Allosteric modulator, Antagonist), [^35^S]TBPS [anion channel] (Channel blocker), [^3^H]CGS8216 [benzodiazepine site] (Allosteric modulator, Mixed), [^3^H]flunitrazepam [benzodiazepine site] (Allosteric modulator, Full agonist), [^3^H]gabazine [GABA site] (Antagonist), [^3^H]muscimol [GABA site] (Agonist)	[^11^C]flumazenil [benzodiazepine site] (Allosteric modulator, Partial agonist), [^18^F]fluoroethylflumazenil [benzodiazepine site] (Allosteric modulator, Antagonist), [^35^S]TBPS [anion channel] (Channel blocker), [^3^H]CGS8216 [benzodiazepine site] (Allosteric modulator, Mixed), [^3^H]Ro154513 [benzodiazepine site] (Allosteric modulator, Full agonist), [^3^H]gabazine [GABA site] (Antagonist), [^3^H]muscimol [GABA site] (Agonist)
Comments	Zn^2+^ is an endogenous allosteric regulator and causes potent inhibition of receptors formed from binary combinations of *α* and *β* subunits, incorporation of a *δ* or *γ* subunit causes a modest, or pronounced, reduction in inhibitory potency, respectively [208]	diazepam and flunitrazepam are not active at this subunit. Zn^2+^ is an endogenous allosteric regulator and causes potent inhibition of receptors formed from binary combinations of *α* and *β* subunits, incorporation of a *δ* or *γ* subunit causes a modest, or pronounced, reduction in inhibitory potency, respectively [208]. [^3^H]Ro154513 selectively labels *α*4‐subunit‐containing receptors in the presence of a saturating concentration of a 'classical' benzodiazepine (e.g. diazepam)

**Table:** 

Nomenclature	GABA_A_ receptor *α*5 subunit	GABA_A_ receptor *α*6 subunit
HGNC, UniProt	GABRA5, P31644	GABRA6, Q16445
Agonists	gaboxadol [GABA site], isoguvacine [GABA site], isonipecotic acid [GABA site], muscimol [GABA site], piperidine‐4‐sulphonic acid [GABA site]	gaboxadol [GABA site], isoguvacine [GABA site], muscimol [GABA site], piperidine‐4‐sulphonic acid [GABA site] (low efficacy)
Selective agonists	–	isonipecotic acid [GABA site] (relatively high efficacy)
Selective antagonists	bicuculline [GABA site], gabazine [GABA site]	bicuculline [GABA site], gabazine [GABA site]
Channel blockers	TBPS, picrotoxin	TBPS, picrotoxin
Endogenous allosteric modulators	5*α*‐pregnan‐3*α*‐ol‐20‐one (Potentiation), Zn^2+^ (Inhibition), tetrahydrodeoxycorticosterone (Potentiation)	5*α*‐pregnan‐3*α*‐ol‐20‐one (Potentiation), Zn^2+^ (Inhibition), tetrahydrodeoxycorticosterone (Potentiation)
Allosteric modulators	flumazenil [benzodiazepine site] (Antagonist) (p*K*_i_ 9.2) [159], flunitrazepam [benzodiazepine site] (Positive) (p*K*_i_ 8.3) [137], alprazolam [benzodiazepine site] (Positive) (pEC_50_ 8) [5], *α*3IA [benzodiazepine site] (Inverse agonist), DMCM [benzodiazepine site] (Inverse agonist)	flumazenil [benzodiazepine site] (Partial agonist) (p*K*_i_ 6.8) [159], bretazenil [benzodiazepine site] (Full agonist)
Selective allosteric modulators	*α*5IA [benzodiazepine site] (Inverse agonist), L655708 [benzodiazepine site] (Inverse agonist), L838417 [benzodiazepine site] (Partial agonist), MRK016 [benzodiazepine site] (Inverse agonist), RO4938581 [benzodiazepine site] (Inverse agonist), RY024 [benzodiazepine site] (Inverse agonist)	Ro15‐4513 [benzodiazepine site] (Full agonist)
Labelled ligands	[^3^H]RY80 [benzodiazepine site] (Selective Binding) (p*K*_d_ 9.2) [335] – Rat, [^11^C]flumazenil [benzodiazepine site] (Allosteric modulator, Antagonist), [^18^F]fluoroethylflumazenil [benzodiazepine site] (Allosteric modulator, Antagonist), [^35^S]TBPS [anion channel] (Channel blocker), [^3^H]CGS8216 [benzodiazepine site] (Allosteric modulator, Mixed), [^3^H]L655708 [benzodiazepine site] (Allosteric modulator, Inverse agonist), [^3^H]flunitrazepam [benzodiazepine site] (Allosteric modulator, Full agonist), [^3^H]gabazine [GABA site] (Antagonist), [^3^H]muscimol [GABA site] (Agonist)	[^11^C]flumazenil [benzodiazepine site] (Allosteric modulator, Partial agonist), [^18^F]fluoroethylflumazenil [benzodiazepine site] (Allosteric modulator, Antagonist), [^35^S]TBPS [anion channel] (Channel blocker), [^3^H]CGS8216 [benzodiazepine site] (Allosteric modulator, Mixed), [^3^H]Ro154513 [benzodiazepine site] (Allosteric modulator, Full agonist), [^3^H]gabazine [GABA site] (Antagonist), [^3^H]muscimol [GABA site] (Agonist)
Comments	Zn^2+^ is an endogenous allosteric regulator and causes potent inhibition of receptors formed from binary combinations of *α* and *β* subunits, incorporation of a *δ* or *γ* subunit causes a modest, or pronounced, reduction in inhibitory potency, respectively [208]	diazepam and flunitrazepam are not active at this subunit. Zn^2+^ is an endogenous allosteric regulator and causes potent inhibition of receptors formed from binary combinations of *α* and *β* subunits, incorporation of a *δ* or *γ* subunit causes a modest, or pronounced, reduction in inhibitory potency, respectively [208]. [^3^H]Ro154513 selectively labels *α*6‐subunit‐containing receptors in the presence of a saturating concentration of a 'classical' benzodiazepine (e.g. diazepam)

**Table:** 

Nomenclature	GABA_A_ receptor *β*1 subunit	GABA_A_ receptor *β*2 subunit	GABA_A_ receptor *β*3 subunit
HGNC, UniProt	GABRB1, P18505	GABRB2, P47870	GABRB3, P28472
Channel blockers	TBPS, picrotoxin	TBPS, picrotoxin	TBPS, picrotoxin
Allosteric modulators	–	–	etazolate (Binding) (pIC_50_ 5.5) [404]
Comments	Zn^2+^ is an endogenous allosteric regulator and causes potent inhibition of receptors formed from binary combinations of *α* and *β* subunits, incorporation of a *δ* or *γ* subunit causes a modest, or pronounced, reduction in inhibitory potency, respectively [208]

**Table:** 

Nomenclature	GABA_A_ receptor *γ*1 subunit	GABA_A_ receptor *γ*2 subunit	GABA_A_ receptor *γ*3 subunit
HGNC, UniProt	GABRG1, Q8N1C3	GABRG2, P18507	GABRG3, Q99928
Channel blockers	TBPS, picrotoxin	TBPS, picrotoxin	TBPS, picrotoxin
Comments	Zn^2+^ is an endogenous allosteric regulator and causes potent inhibition of receptors formed from binary combinations of *α* and *β* subunits, incorporation of a *δ* or *γ* subunit causes a modest, or pronounced, reduction in inhibitory potency, respectively [208]

**Table:** 

Nomenclature	GABA_A_ receptor *δ* subunit	GABA_A_ receptor *ϵ* subunit	GABA_A_ receptor *θ* subunit	GABA_A_ receptor *π* subunit
HGNC, UniProt	GABRD, O14764	GABRE, P78334	GABRQ, Q9UN88	GABRP, O00591
Selective agonists	gaboxadol [GABA site]	–	–	–
Channel blockers	TBPS, picrotoxin	TBPS, picrotoxin	TBPS, picrotoxin	TBPS, picrotoxin
Comments	Zn^2+^ is an endogenous allosteric regulator and causes potent inhibition of receptors formed from binary combinations of *α* and *β* subunits, incorporation of a *δ* or *γ* subunit causes a modest, or pronounced, reduction in inhibitory potency, respectively	–	–	–

**Table:** 

Nomenclature	GABA_A_ receptor *ρ*1 subunit	GABA_A_ receptor *ρ*2 subunit	GABA_A_ receptor *ρ*3 subunit
HGNC, UniProt	GABRR1, P24046	GABRR2, P28476	GABRR3, A8MPY1
Agonists	isoguvacine [GABA site] (Partial agonist), muscimol [GABA site] (Partial agonist)	isoguvacine [GABA site] (Partial agonist), muscimol [GABA site] (Partial agonist)	isoguvacine [GABA site] (Partial agonist), muscimol [GABA site] (Partial agonist)
Selective agonists	(±)‐cis‐2‐CAMP [GABA site], 5‐Me‐IAA [GABA site]	(±)‐cis‐2‐CAMP [GABA site], 5‐Me‐IAA [GABA site]	(±)‐cis‐2‐CAMP [GABA site], 5‐Me‐IAA [GABA site]
Antagonists	gaboxadol [GABA site], isonipecotic acid [GABA site], piperidine‐4‐sulphonic acid [GABA site]	gaboxadol [GABA site], isonipecotic acid [GABA site], piperidine‐4‐sulphonic acid [GABA site]	gaboxadol [GABA site], isonipecotic acid [GABA site], piperidine‐4‐sulphonic acid [GABA site]
Selective antagonists	cis‐3‐ACPBPA [GABA site], trans‐3‐ACPBPA [GABA site], TPMPA [GABA site], aza‐THIP [GABA site]	cis‐3‐ACPBPA [GABA site], trans‐3‐ACPBPA [GABA site], TPMPA [GABA site], aza‐THIP [GABA site]	cis‐3‐ACPBPA [GABA site], trans‐3‐ACPBPA [GABA site], TPMPA [GABA site], aza‐THIP [GABA site]
Channel blockers	TBPS, picrotoxin	TBPS, picrotoxin	TBPS, picrotoxin
Comments	bicuculline is not active at this subunit	bicuculline is not active at this subunit	bicuculline is not active at this subunit

### Comments

The potency and efficacy of many GABA agonists vary between GABA_A_ receptor isoforms [117, 188, 209]. For example, gaboxadol is a partial agonist at receptors with the subunit composition *α*4*β*3*γ*2, but elicits currents in excess of those evoked by GABA at the *α*4*β*3*δ* receptor where GABA itself is a low efficacy agonist [32, 43]. The antagonists bicuculline and gabazine differ in their ability to suppress spontaneous openings of the GABA_A_ receptor, the former being more effective [359]. The presence of the *γ* subunit within the heterotrimeric complex reduces the potency and efficacy of agonists [347]. The GABA_A_ receptor contains distinct allosteric sites that bind barbiturates and endogenous (e.g., 5*α*‐pregnan‐3*α*‐ol‐20‐one) and synthetic (e.g., alphaxalone) neuroactive steroids in a diastereo‐ or enantio‐selective manner [28, 143, 154, 368]. Picrotoxinin and TBPS act at an allosteric site within the chloride channel pore to negatively regulate channel activity; negative allosteric regulation by *γ*‐butyrolactone derivatives also involves the picrotoxinin site, whereas positive allosteric regulation by such compounds is proposed to occur at a distinct locus. Many intravenous (e.g., etomidate, propofol) and inhalational (e.g., halothane, isoflurane) anaesthetics and alcohols also exert a regulatory influence upon GABA_A_ receptor activity [38, 285]. Specific amino acid residues within GABA_A_ receptor *α*‐ and *β*‐subunits that influence allosteric regulation by anaesthetic and non‐anaesthetic compounds have been identified [141, 154]. Photoaffinity labelling of distinct amino acid residues within purified GABA_A_receptors by the etomidate derivative, [^3^H]azietomidate, has also been demonstrated [221] and this binding subject to positive allosteric regulation by anaesthetic steroids [220]. An array of natural products including flavonoid and terpenoid compounds exert varied actions at GABA_A_receptors (reviewed in detail in [181]).

In addition to the agents listed in the table, modulators of GABA_A_ receptor activity that exhibit subunit dependent activity include: salicylidene salicylhydrazide [negative allosteric modulator selective for *β*1‐ versus *β*2‐, or *β*3‐subunit‐containing receptors [360]]; fragrent dioxane derivatives [positive allosteric modulators selective for *β*1‐ versus *β*2‐, or *β*3‐subunit‐containing receptors [328]]; loreclezole, etomidate, tracazolate, mefenamic acid, etifoxine, stiripentol, valerenic acid amide [positive allosteric modulators with selectivity for *β*2/*β*3‐ over *β*1‐subunit‐containing receptors [112, 198, 206]]; tracazolate[intrinsic efficacy, i.e., potentiation, or inhibition, is dependent upon the identity of the *γ*1‐3‐, *δ*‐, or *ϵ*‐subunit co‐assembed with *α*1‐ and *β*1‐subunits [358]]; amiloride [selective blockade of receptors containing an *α*6‐subunit [115]]; furosemide [selective blockade of receptors containing an *α*6‐subunit co‐assembled with *β*2/*β*3‐, but not *β*1‐subunit [206]]; La^3+^[potentiates responses mediated by *α*1*β*3*γ*2L receptors, weakly inhibits *α*6*β*3*γ*2L receptors, and strongly blocks *α*6*β*3*δ* and *α*4*β*3*δ* receptors [43, 321]]; ethanol [selectively potentiates responses mediated by *α*4*β*3*δ* and *α*6*β*3*δ* receptors versus receptors in which *β*2 replaces *β*3, or *γ*replaces *δ*[378], but see also [205]]; DS1 and DS2 [selectively potentiate responses mediated by *δ*‐subunit‐containing receptors [374]]. It should be noted that the apparent selectivity of some positive allosteric modulators (e.g., neurosteroids such as 5*α*‐pregnan‐3*α*‐ol‐20‐one for *δ*‐subunit‐containing receptors (e.g., *α*1*β*3*δ*) may be a consequence of the unusually low efficacy of GABA at this receptor isoform [27, 32].

### Further reading on GABA_A_ receptors

Braat, S et al. (2015) The GABAA Receptor as a Therapeutic Target for Neurodevelopmental Disorders. Neuron 86: 1119‐30 [PMID:26050032]

Calvo, DJ et al. (2016) Dynamic Regulation of the GABAA Receptor Function by Redox Mechanisms. Mol Pharmacol 90: 326‐33 [PMID:27439531]

Locci, A et al. (2017) Neurosteroid biosynthesis downregulation and changes in GABAA receptor subunit composition: A biomarker axis in stress‐induced cognitive and emotional impairment. Br J Pharmacol [PMID:28456011]

Mele, M et al. (2016) Role of GABAA R trafficking in the plasticity of inhibitory synapses. J Neurochem 139: 997‐1018 [PMID:27424566]

Olsen, RW et al. (2008) International Union of Pharmacology. LXX. Subtypes of gamma‐aminobutyric acid(A) receptors: classification on the basis of subunit composition, pharmacology, and function. Update. Pharmacol Rev 60: 243‐60 [PMID:18790874]

## Glycine receptors

### Overview

The inhibitory glycine receptor (**nomenclature as agreed by the NC‐IUPHAR Subcommittee on Glycine Receptors**) is a member of the Cys‐loop superfamily of transmitter‐gated ion channels that includes the zinc activated channels, GABA_A_, nicotinic acetylcholine and 5‐HT_3_ receptors [233]. The receptor is expressed either as a homo‐pentamer of *α*subunits, or a complex now thought to harbour 2*α* and 3*β* subunits [30, 129], that contain an intrinsic anion channel. Four differentially expressed isoforms of the *α*‐subunit (*α*1‐*α*4) and one variant of the *β*‐subunit (*β*1, GLRB, P48167) have been identified by genomic and cDNA cloning. Further diversity originates from alternative splicing of the primary gene transcripts for *α*1 (*α*1^INS^ and *α*1^del^), *α*2 (*α*2A and *α*2B), *α*3 (*α*3S and *α*3L) and *β*(*β*Δ7) subunits and by mRNA editing of the *α*2 and *α*3 subunit [103, 249, 284]. Both *α*2 splicing and *α*3 mRNA editing can produce subunits (i.e., *α*2B and *α*3P185L) with enhanced agonist sensitivity. Predominantly, the mature form of the receptor contains *α*1 (or *α*3) and *β* subunits while the immature form is mostly composed of only *α*2 subunits. RNA transcripts encoding the *α*4‐subunit have not been detected in adult humans. The N‐terminal domain of the *α*‐subunit contains both the agonist and strychnine binding sites that consist of several discontinuous regions of amino acids. Inclusion of the *β*‐subunit in the pentameric glycine receptor contributes to agonist binding, reduces single channel conductance and alters pharmacology. The *β*‐subunit also anchors the receptor, via an amphipathic sequence within the large intracellular loop region, to gephyrin. The latter is a cytoskeletal attachment protein that binds to a number of subsynaptic proteins involved in cytoskeletal structure and thus clusters and anchors hetero‐oligomeric receptors to the synapse [201, 204, 268]. G‐protein *β* *γ* subunits enhance the open state probability of native and recombinant glycine receptors by association with domains within the large intracellular loop [397, 398]. Intracellular chloride concentration modulates the kinetics of native and recombinant glycine receptors [304]. Intracellular Ca^2+^ appears to increase native and recombinant glycine receptor affinity, prolonging channel open events, by a mechanism that does not involve phosphorylation [118].

**Table:** 

Nomenclature	glycine receptor *α*1 subunit	glycine receptor *α*2 subunit	glycine receptor *α*3 subunit	glycine receptor *α*4 subunit (pseudogene in humans)
HGNC, UniProt	GLRA1, P23415	GLRA2, P23416	GLRA3, O75311	GLRA4, Q5JXX5
Selective agonists (potency order)	glycine>*β*‐alanine>taurine	glycine>*β*‐alanine>taurine	glycine>*β*‐alanine>taurine	–
Selective antagonists	ginkgolide X (pIC_50_ 6.1), pregnenolone sulphate (p*K*_i_ 5.7), nifedipine (pIC_50_ 5.5), bilobalide (pIC_50_ 4.7), tropisetron (p*K*_i_ 4.1), colchicine (pIC_50_ 3.5), HU‐308 (weak inhibition), PMBA, strychnine	HU‐210 (pIC_50_ 7), WIN55212‐2 (pIC_50_ 6.7), HU‐308 (pIC_50_ 6), ginkgolide X (pIC_50_ 5.6), pregnenolone sulphate (p*K*_i_ 5.3), bilobalide (pIC_50_ 5.1), tropisetron (p*K*_i_ 4.9), colchicine (pIC_50_ 4.2), 5,7‐dichlorokynurenic acid (pIC_50_ 3.7), PMBA, strychnine	HU‐210 (pIC_50_ 7.3), HU‐308 (pIC_50_ 7), WIN55212‐2 (pIC_50_ 7), (12E,20Z,18S)‐8‐hydroxyvariabilin (pIC_50_ 5.2), nifedipine (pIC_50_ 4.5), strychnine	–
Channel blockers	ginkgolide B (pIC_50_ 5.1–6.2), cyanotriphenylborate (pIC_50_ 5.9) [316], picrotin (pIC_50_ 5.3), picrotoxinin (pIC_50_ 5.3), picrotoxin (pIC_50_ 5.2)	picrotoxinin (pIC_50_ 6.4), picrotoxin (pIC_50_ 5.6), ginkgolide B (pIC_50_ 4.9–5.4), picrotin (pIC_50_ 4.9), cyanotriphenylborate (pIC_50_>4.7) [316]	picrotoxinin (pIC_50_ 6.4), ginkgolide B (pIC_50_ 5.7), picrotin (pIC_50_ 5.2), picrotoxin (block is weaker when *β* subunit is co‐expressed)	–
Endogenous allosteric modulators	Zn^2+^ (Potentiation) (pEC_50_ 7.4), Cu^2+^ (Inhibition) (pIC_50_ 4.8–5.4), Zn^2+^ (Inhibition) (pIC_50_ 4.8), Extracellular H^+^ (Inhibition)	Zn^2+^ (Potentiation) (pEC_50_ 6.3), Cu^2+^ (Inhibition) (pIC_50_ 4.8), Zn^2+^ (Inhibition) (pIC_50_ 3.4)	Cu^2+^ (Inhibition) (pIC_50_ 5), Zn^2+^ (Inhibition) (pIC_50_ 3.8)	–
Selective allosteric modulators	anandamide (Potentiation) (pEC_50_ 7.4), HU‐210 (Potentiation) (pEC_50_ 6.6), Δ^9^‐tetrahydrocannabinol (Potentiation) (pEC_50_∼5.5)	Δ^9^‐tetrahydrocannabinol (Potentiation) (pEC_50_∼6)	Δ^9^‐tetrahydrocannabinol (Potentiation) (pEC_50_∼5.3)	–
Labelled ligands	[^3^H]strychnine (Antagonist)	[^3^H]strychnine (Antagonist)	[^3^H]strychnine (Antagonist)	–
Functional Characteristics	*γ* = 86 pS (main state); (+ *β* = 44 pS)	*γ* = 111 pS (main state); (+ *β* = 54 pS)	*γ* = 105 pS (main state); (+ *β* = 48)	–

**Table:** 

Nomenclature	glycine receptor *β* subunit
HGNC, UniProt	GLRB, P48167
Selective antagonists	nifedipine (when co‐expressed with the *α*1 subunit) (pIC_50_ 5.9), pregnenolone sulphate (when co‐expressed with the *α*1 subunit) (p*K*_i_ 5.6), tropisetron (when co‐expressed with the *α*2 subunit) (p*K*_i_ 5.3), pregnenolone sulphate (when co‐expressed with the *α*2 subunit) (p*K*_i_ 5), nifedipine (when co‐expressed with the *α*3 subunit) (pIC_50_ 4.9), bilobalide (when co‐expressed with the *α*2 subunit) (pIC_50_ 4.3), bilobalide (when co‐expressed with the *α*1 subunit) (pIC_50_ 3.7), ginkgolide X (when co‐expressed with the *α*1 subunit) (pIC_50_>3.5), ginkgolide X (when co‐expressed with the *α*2 subunit) (pIC_50_>3.5)
Channel blockers	ginkgolide B (when co‐expressed with the *α*2 subunit) (pIC_50_ 6.1–6.9), ginkgolide B (when co‐expressed with the *α*1 subunit) (pIC_50_ 5.6–6.7), ginkgolide B (when co‐expressed with the *α*3 subunit) (pIC_50_ 6.3), cyanotriphenylborate (when co‐expressed with the human *α*1 subunit) (pIC_50_ 5.6) [316] – Rat, cyanotriphenylborate (when co‐expressed with the human *α*2 subunit) (pIC_50_ 5.1) [316] – Rat, picrotoxinin (when co‐expressed with the *α*3 subunit) (pIC_50_ 5.1), picrotin (when co‐expressed with the *α*1 subunit) (pIC_50_ 4.6), picrotin (when co‐expressed with the *α*3 subunit) (pIC_50_ 4.6), picrotoxinin (when co‐expressed with the *α*1 subunit) (pIC_50_ 4.6), picrotoxin (when co‐expressed with the *α*2 subunit) (pIC_50_ 4.5), picrotoxin (when co‐expressed with the *α*1 subunit) (pIC_50_ 3.7)
Endogenous allosteric modulators	Zn^2+^ (Inhibition) (pIC_50_ 4.9), Zn^2+^ (Inhibition) (pIC_50_ 3.7)
Comments	Ligand interaction data for hetero‐oligomer receptors containing the *β* subunit are also listed under the *α* subunit

### Comments

Data in the table refer to homo‐oligomeric assemblies of the *α*‐subunit, significant changes introduced by co‐expression of the *β*1 subunit are indicated in parenthesis. Not all glycine receptor ligands are listed within the table, but some that may be useful in distinguishing between glycine receptor isoforms are indicated (see detailed view pages for each subunit: *α*1, *α*2, *α*3, *α*4, *β* ). Pregnenolone sulphate, tropisetron and colchicine, for example, although not selective antagonists of glycine receptors, are included for this purpose. Strychnine is a potent and selective competitive glycine receptor antagonist with affinities in the range 5–15 nM. RU5135 demonstrates comparable potency, but additionally blocks GABA_A_ receptors. There are conflicting reports concerning the ability of cannabinoids to inhibit [228], or potentiate and at high concentrations activate [3, 83, 140, 389, 394] glycine receptors. Nonetheless, cannabinoid analogues may hold promise in distinguishing between glycine receptor subtypes [394]. In addition, potentiation of glycine receptor activity by cannabinoids has been claimed to contribute to cannabis‐induced analgesia relying on Ser296/307 (*α*1/*α*3) in M3 [389]. Several analogues of muscimol and piperidine act as agonists and antagonists of both glycine and GABA_A_receptors. Picrotoxin acts as an allosteric inhibitor that appears to bind within the pore, and shows strong selectivity towards homomeric receptors. While its components, picrotoxinin and picrotin, have equal potencies at *α*1 receptors, their potencies at *α*2 and *α*3 receptors differ modestly and may allow some distinction between different receptor types [395]. Binding of picrotoxin within the pore has been demonstrated in the crystal structure of the related C. elegans GluCl Cys‐loop receptor [144]. In addition to the compounds listed in the table, numerous agents act as allosteric regulators of glycine receptors (comprehensively reviewed in [215, 234, 381, 399]). Zn^2+^ acts through distinct binding sites of high‐ and low‐affinity to allosterically enhance channel function at low (<10 μM) concentrations and inhibits responses at higher concentrations in a subunit selective manner [258]. The effect of Zn^2+^ is somewhat mimicked by Ni^2+^. Endogenous Zn^2+^ is essential for normal glycinergic neurotransmission mediated by *α*1 subunit‐containing receptors [147]. Elevation of intracellular Ca^2+^ produces fast potentiation of glycine receptor‐mediated responses. Dideoxyforskolin (4 μM) and tamoxifen (0.2–5 μM) both potentiate responses to low glycine concentrations (15 μM), but act as inhibitors at higher glycine concentrations (100 μM). Additional modulatory agents that enhance glycine receptor function include inhalational, and several intravenous general anaesthetics (e.g. minaxolone, propofol and pentobarbitone) and certain neurosteroids. Ethanol and higher order n‐alcohols also enhance glycine receptor function although whether this occurs by a direct allosteric action at the receptor [245], or through *β* *γ* subunits [396] is debated. Recent crystal structures of the bacterial homologue, GLIC, have identified transmembrane binding pockets for both anaesthetics [282] and alcohols [156]. Solvents inhaled as drugs of abuse (e.g. toluene, 1‐1‐1‐trichloroethane) may act at sites that overlap with those recognising alcohols and volatile anaesthetics to produce potentiation of glycine receptor function. The function of glycine receptors formed as homomeric complexes of *α*1 or *α*2 subunits, or hetero‐oligomers of *α*1/*β* or *α*2/*β* subunits, is differentially affected by the 5‐HT_3_ receptor antagonist tropisetron (ICS 205‐930) which may evoke potentiation (which may occur within the femtomolar range at the homomeric glycine *α*1 receptor), or inhibition, depending upon the subunit composition of the receptor and the concentrations of the modulator and glycine employed. Potentiation and inhibition by tropeines involves different binding modes [238]. Additional tropeines, including atropine, modulate glycine receptor activity.

### Further reading on Glycine receptors

Betz, H et al. (2006) Glycine receptors: recent insights into their structural organization and functional diversity. J Neurochem 97: 1600‐10 [PMID:16805771]

Burgos, CF et al. (2016) Structure and Pharmacologic Modulation of Inhibitory Glycine Receptors. Mol Pharmacol 90: 318‐25 [PMID:27401877]

Dutertre, S et al. (2012) Inhibitory glycine receptors: an update. J Biol Chem 287: 40216‐23 [PMID:23038260]

Lynch, JW. (2004) Molecular structure and function of the glycine receptor chloride channel. Physiol Rev 84: 1051‐95 [PMID:15383648]

Perkins, DI et al. (2010) Molecular targets and mechanisms for ethanol action in glycine receptors. Pharmacol Ther 127: 53‐65 [PMID:20399807]

Yevenes, GE et al. (2011) Allosteric modulation of glycine receptors. Br J Pharmacol 164: 224‐36 [PMID:21557733]

## Ionotropic glutamate receptors

### Overview

The ionotropic glutamate receptors comprise members of the NMDA (N‐methyl‐D‐aspartate), AMPA (*α*‐amino‐3‐hydroxy‐5‐methyl‐4‐isoxazoleproprionic acid) and kainate receptor classes, named originally according to their preferred, synthetic, agonist [86, 226, 365]. Receptor heterogeneity within each class arises from the homo‐oligomeric, or hetero‐oligomeric, assembly of distinct subunits into cation‐selective tetramers. Each subunit of the tetrameric complex comprises an extracellular amino terminal domain (ATD), an extracellular ligand binding domain (LBD), three transmembrane domains composed of three membrane spans (M1, M3 and M4), a channel lining re‐entrant ‘p‐loop’ (M2) located between M1 and M3 and an intracellular carboxy‐ terminal domain (CTD) [184, 211, 246, 271, 365]. The X‐ray structure of a homomeric ionotropic glutamate receptor (GluA2 – see below) has recently been solved at 3.6Å resolution [340] and although providing the most complete structural information current available may not representative of the subunit arrangement of, for example, the heteromeric NMDA receptors [187]. It is beyond the scope of this supplement to discuss the pharmacology of individual ionotropic glutamate receptor isoforms in detail; such information can be gleaned from [62, 74, 86, 106, 171, 176, 195, 288, 289, 290, 365, 388]. Agents that discriminate between subunit isoforms are, where appropriate, noted in the tables and additional compounds that distinguish between receptor isoforms are indicated in the text below.

**The classification of glutamate receptor subunits has been re‐addressed by NC‐IUPHAR**[71]. The scheme developed recommends a nomenclature for ionotropic glutamate receptor subunits that is adopted here.

AMPA and Kainate receptors

AMPA receptors assemble as homomers, or heteromers, that may be drawn from GluA1, GluA2, GluA3 and GluA4 and GluD1, GluD2, GluK1, GluK2, GluK3, GluK4 and GluK5 subunits. Transmembrane AMPA receptor regulatory proteins (TARPs) of class I (i.e. *γ*2, *γ*3, *γ*4 and *γ*8) act, with variable stoichiometry, as auxiliary subunits to AMPA receptors and influence their trafficking, single channel conductance gating and pharmacology (reviewed in [108, 165, 260, 362]). Functional kainate receptors can be expressed as homomers of GluK1, GluK2 or GluK3 subunits. GluK1‐3 subunits are also capable of assembling into heterotetramers (e.g. GluK1/K2; [218, 300, 303]). Two additional kainate receptor subunits, GluK4 and GluK5, when expressed individually, form high affinity binding sites for kainate, but lack function, but can form heteromers when expressed with GluK1‐3 subunits (e.g. GluK2/K5; reviewed in [171, 300, 303]). Kainate receptors may also exhibit ‘metabotropic’ functions [218, 312]. As found for AMPA receptors, kainate receptors are modulated by auxiliary subunits (Neto proteins, [219, 300]). An important function difference between AMPA and kainate receptors is that the latter require extracellular Na+ and Cl‐ for their activation [40, 306]. RNA encoding the GluA2 subunit undergoes extensive RNA editing in which the codon encoding a p‐loop glutamine residue (Q) is converted to one encoding arginine (R). This Q/R site strongly influences the biophysical properties of the receptor. Recombinant AMPA receptors lacking RNA edited GluA2 subunits are: (1) permeable to Ca^2+^; (2) blocked by intracellular polyamines at depolarized potentials causing inward rectification (the latter being reduced by TARPs); (3) blocked by extracellular argiotoxin and Joro spider toxins and (4) demonstrate higher channel conductances than receptors containing the edited form of GluA2 [163, 326]. GluK1 and GluK2, but not other kainate receptor subunits, are similarly edited and broadly similar functional characteristics apply to kainate receptors lacking either an RNA edited GluK1, or GluK2, subunit [218, 300]. Native AMPA and kainate receptors displaying differential channel conductances, Ca^2+^ permeabilites and sensitivity to block by intracellular polyamines have been identified [73, 163, 224]. GluA1‐4 can exist as two variants generated by alternative splicing (termed ‘flip’ and ‘flop’) that differ in their desensitization kinetics and their desensitization in the presence of cyclothiazide which stabilises the nondesensitized state. TARPs also stabilise the non‐desensitized conformation of AMPA receptors and facilitate the action of cyclothiazide[260]. Splice variants of GluK1‐3 also exist which affects their trafficking [218, 300].

**Table:** 

Nomenclature	GluA1	GluA2	GluA3	GluA4
HGNC, UniProt	GRIA1, P42261	GRIA2, P42262	GRIA3, P42263	GRIA4, P48058
Agonists	(S)‐5‐fluorowillardiine, AMPA	(S)‐5‐fluorowillardiine, AMPA	(S)‐5‐fluorowillardiine, AMPA	(S)‐5‐fluorowillardiine, AMPA
Selective antagonists	ATPO, GYKI53655, GYKI53784 (active isomer, non‐competitive), NBQX, tezampanel	ATPO, GYKI53655, GYKI53784 (active isomer, non‐competitive), NBQX, tezampanel	ATPO, GYKI53655, GYKI53784 (active isomer, non‐competitive), NBQX, tezampanel	ATPO, GYKI53655, GYKI53784 (active isomer, non‐competitive), NBQX, tezampanel
Channel blockers	extracellular argiotoxin, extracellular joro toxin (selective for channels lacking GluA2)	extracellular argiotoxin	extracellular argiotoxin, extracellular joro toxin (selective for channels lacking GluA2)	extracellular argiotoxin, extracellular joro toxin (selective for channels lacking GluA2)
Allosteric modulators	LY392098 (Positive) (pEC_50_ 5.8) [261], LY404187 (Positive) (pEC_50_ 5.2) [261], cyclothiazide (Positive) (pEC_50_ 4.7) [261], CX516 (Positive), CX546 (Positive), IDRA‐21 (Positive), LY503430 (Positive), S18986 (Positive), aniracetam (Positive), piracetam (Positive)	LY404187 (Positive) (pEC_50_ 6.8) [261], LY392098 (Positive) (pEC_50_ 6.7) [261], cyclothiazide (Positive) (pEC_50_ 5.7) [261], CX516 (Positive), CX546 (Positive), IDRA‐21 (Positive), LY503430 (Positive), S18986 (Positive), aniracetam (Positive), piracetam (Positive)	LY404187 (Positive) (pEC_50_ 5.8) [261], LY392098 (Positive) (pEC_50_ 5.7) [261], cyclothiazide (Positive) (pEC_50_ 4.9) [261], CX516 (Positive), CX546 (Positive), IDRA‐21 (Positive), LY503430 (Positive), S18986 (Positive), aniracetam (Positive), piracetam (Positive)	LY392098 (Positive) (pEC_50_ 6.7) [261], LY404187 (Positive) (pEC_50_ 6.7) [261], cyclothiazide (Positive) (pEC_50_ 5.4) [261], CX516 (Positive), CX546 (Positive), IDRA‐21 (Positive), LY503430 (Positive), S18986 (Positive), aniracetam (Positive), piracetam (Positive)
Labelled ligands	[^3^H]AMPA (Agonist), [^3^H]CNQX (Antagonist)	[^3^H]AMPA (Agonist), [^3^H]CNQX (Antagonist)	[^3^H]AMPA, [^3^H]CNQX	[^3^H]AMPA (Agonist), [^3^H]CNQX
Comments	Also blocked by intracellular polyamines.	Also blocked by intracellular polyamines.	Also blocked by intracellular polyamines	Also blocked by intracellular polyamines

**Table:** 

Nomenclature	GluD1	GluD2
HGNC, UniProt	GRID1, Q9ULK0	GRID2, O43424

**Table:** 

Nomenclature	GluK1	GluK2	GluK3	GluK4	GluK5
HGNC, UniProt	GRIK1, P39086	GRIK2, Q13002	GRIK3, Q13003	GRIK4, Q16099	GRIK5, Q16478
Endogenous agonists			–	–	–
Agonists	dysiherbaine [320] – Rat, SYM2081 [296], kainate [336], (S)‐4‐AHCP, (S)‐5‐iodowillardiine, 8‐deoxy‐neodysiherbaine, ATPA, domoic acid	dysiherbaine [320] – Rat, domoic acid [50], SYM2081 [413] – Rat, kainate [50, 336]	SYM2081 [318] – Rat, kainate (low potency) [318] – Rat, dysiherbaine	SYM2081, domoic acid, dysiherbaine, kainate	SYM2081, domoic acid, dysiherbaine, kainate
Selective agonists	LY339434 [342]	–			
Selective antagonists	2,4‐epi‐neodysiherbaine, ACET, LY382884, LY466195, MSVIII‐19, NS3763 (non‐competitive), UBP302, UBP310	2,4‐epi‐neo dysiherbaine	–	–	–
Allosteric modulators	concanavalin A (Positive)	concanavalin A (Positive)	–	–	–
Labelled ligands	[^3^H]UBP310 (Antagonist) (p*K*_d_ 7.7) [13], [^3^H]SYM2081 (Agonist), [^3^H]kainate (Agonist)	[^3^H]kainate (Agonist) [407] – Rat, [^3^H]SYM2081 (Agonist)	[^3^H]UBP310 (Antagonist) (p*K*_d_ 6.3) [13], [^3^H]SYM2081 (Agonist), [^3^H]kainate (Agonist)	[^3^H]SYM2081 (Agonist), [^3^H]kainate (Agonist)	[^3^H]SYM2081 (Agonist), [^3^H]kainate (Agonist)
Comments	–	Intracellular polyamines are subtype selective channel blockers (GluK3 ≫GluK2)	domoic acid and concanavalin A are inactive at the GluK3 subunit. Intracellular polyamines are subtype selective channel blockers (GluK3 ≫GluK2)	–	–

### NMDA receptors

NMDA receptors assemble as obligate heteromers that may be drawn from GluN1, GluN2, GluN3 and GluN4 subunits. Alternative splicing can generate eight isoforms of GluN1 with differing pharmacological properties. Various splice variants of GluN2B, 2C, 2D and GluN3A have also been reported. Activation of NMDA receptors containing GluN1 and GluN2 subunits requires the binding of two agonists, glutamate to the S1 and S2 regions of the GluN2 subunit and glycine to S1 and S2 regions of the GluN1 subunit [63, 105]. The minimal requirement for efficient functional expression of NMDA receptors in vitro is a di‐heteromeric assembly of GluN1 and at least one GluN2 subunit variant, as a dimer of heterodimers arrangement in the extracellular domain [119, 187, 246]. However, more complex tri‐heteromeric assemblies, incorporating multiple subtypes of GluN2 subunit, or GluN3 subunits, can be generatedin vitro and occurin vivo. The NMDA receptor channel commonly has a high relative permeability to Ca^2+^ and is blocked, in a voltage‐dependent manner, by Mg^2+^ such that at resting potentials the response is substantially inhibited.

**Table:** 

Nomenclature	GluN1	GluN2A	GluN2B	GluN2C	GluN2D
HGNC, UniProt	GRIN1, Q05586	GRIN2A, Q12879	GRIN2B, Q13224	GRIN2C, Q14957	GRIN2D, O15399
Endogenous agonists	D‐aspartic acid [glutamate site], D‐serine [glycine site], L‐aspartic acid [glutamate site], glycine [glycine site]	D‐aspartic acid[glutamate site] (GluN2D > GluN2C = GluN2B > GluN2A), D‐serine[glycine site] (GluN2D > GluN2C > GluN2B > GluN2A), L‐aspartic acid[glutamate site] (GluN2D = GluN2B > GluN2C = GluN2A), glycine[glycine site] (GluN2D > GluN2C > GluN2B > GluN2A)		D‐aspartic acid[glutamate site] (GluN2D > GluN2C = GluN2B > GluN2A), D‐serine[glycine site] (GluN2D > GluN2C > GluN2B > GluN2A), L‐aspartic acid[glutamate site] (GluN2D = GluN2B > GluN2C = GluN2A), glycine[glycine site] (GluN2D > GluN2C > GluN2B > GluN2A)	
Agonists	(+)‐HA966 [glycine site] (Partial agonist), (RS)‐(tetrazol‐5‐yl)glycine [glutamate site], NMDA [glutamate site], homoquinolinic acid [glutamate site] (Partial agonist)	(+)‐HA966[glycine site] (Partial agonist), (RS)‐(tetrazol‐5‐yl)glycine[glutamate site] (GluN2D > GluN2C = GluN2B > GluN2A), NMDA[glutamate site] (GluN2D > GluN2C > GluN2B > GluN2A), homoquinolinic acid[glutamate site] (GluN2B ≥ GluN2A ≥ GluN2D > GluN2C; partial agonist at GluN2A and GluN2C)		(RS)‐(tetrazol‐5‐yl)glycine[glutamate site] (GluN2D > GluN2C = GluN2B > GluN2A), NMDA[glutamate site] (GluN2D > GluN2C > GluN2B > GluN2A), homoquinolinic acid[glutamate site] (GluN2B ≥ GluN2A ≥ GluN2D > GluN2C; partial agonist at GluN2A and GluN2C)	
Selective antagonists	L701324 [glycine site] (pIC_50_ 8.7) [210] – Rat, GV196771A [glycine site] (p*K*_i_ 8.1–8.4) [67] – Rat, L689560 [glycine site] (pIC_50_ 8.1) [217] – Rat, 5,7‐dichlorokynurenic acid [glycine site]	5,7‐dichlorokynurenic acid[glycine site], CGP37849[glutamate site], GV196771A[glycine site], L689560[glycine site], L701324[glycine site], LY233053[glutamate site], NVP‐AAM077[glutamate site] (GluN2A > GluN2B (human), but weakly selective for rat GluN2A versus GluN2B) [14, 110, 116, 273], UBP141[glutamate site] (GluN2D ≥ GluN2C > GluN2A ≥ GluN2B) [266], conantokin‐G[glutamate site] (GluN2B > GluN2D = GluN2C = GluN2A), d‐AP5[glutamate site], d‐CCPene[glutamate site] (GluN2A = GluN2B > GluN2C = GluN2D), selfotel[glutamate site]		5,7‐dichlorokynurenic acid[glycine site], CGP37849[glutamate site], GV196771A[glycine site], L689560[glycine site], L701324[glycine site], LY233053[glutamate site], UBP141[glutamate site] (GluN2D ≥ GluN2C > GluN2A ≥ GluN2B) [266], conantokin‐G[glutamate site] (GluN2B > GluN2D = GluN2C = GluN2A), d‐AP5[glutamate site], d‐CCPene[glutamate site] (GluN2A = GluN2B > GluN2C = GluN2D), selfotel[glutamate site]	
Channel blockers	–	Mg^2+^(GluN2A = GluN2B > GluN2C = GluN2D), N^1^‐dansyl‐spermine(GluN2A = GluN2B ≫ GluN2C = GluN2D), amantidine(GluN2C = GluN2D ≥ GluN2B ≥ GluN2A), dizocilpine, ketamine, phencyclidine		phencyclidine(pIC_50_ 7.1) [96], ketamine(pIC_50_ 6.2) [96], amantidine(GluN2C = GluN2D ≥ GluN2B ≥ GluN2A) (pIC_50_ 4.7) [96], Mg^2+^(GluN2A = GluN2B > GluN2C = GluN2D), N^1^‐dansyl‐spermine(GluN2A = GluN2B ≫ GluN2C = GluN2D), dizocilpine	
Labelled ligands	[^3^H]MDL105519 [glycine site] (Antagonist) (p*K*_d_∼8.5) [59] – Rat, [^3^H]CGP39653 [glutamate site] (Selective Antagonist), [^3^H]CGP61594 [glycine site] (Antagonist), [^3^H]CGS19755 [glutamate site] (Antagonist), [^3^H]CPP [glutamate site] (Selective Antagonist), [^3^H]L689560 [glycine site] (Antagonist), [^3^H]dizocilpine [cation channel] (Antagonist), [^3^H]glycine [glycine site] (Agonist)	[^3^H]CGP39653[glutamate site] (Antagonist), [^3^H]CGP61594[glycine site] (Antagonist), [^3^H]CGS19755[glutamate site] (Antagonist), [^3^H]CPP[glutamate site] (Antagonist), [^3^H]L689560[glycine site] (Antagonist), [^3^H]MDL105519[glycine site] (Antagonist), [^3^H]dizocilpine[cation channel] (Channel blocker), [^3^H]glycine[glycine site] (Agonist)		[^3^H]CGP39653[glutamate site] (Antagonist), [^3^H]CGP61594[glycine site] (Antagonist), [^3^H]CGS19755[glutamate site] (Antagonist), [^3^H]CPP[glutamate site] (Antagonist), [^3^H]L689560[glycine site] (Antagonist), [^3^H]MDL105519[glycine site] (Antagonist), [^3^H]dizocilpine[cation channel] (Channel blocker), [^3^H]glycine[glycine site] (Agonist)

**Table:** 

Nomenclature	GluN3A	GluN3B
HGNC, UniProt	GRIN3A, Q8TCU5	GRIN3B, O60391
Comments	See the main comments section below for information on the pharmacology of GluN3A and GluN3B subunits	

### Comments: NMDA receptors

Potency orders unreferenced in the table are from [62, 96, 106, 212, 290, 365]. In addition to the glutamate and glycine binding sites documented in the table, physiologically important inhibitory modulatory sites exist for Mg^2+^, Zn^2+^, and protons [74, 86, 365]. Voltage‐independent inhibition by Zn^2+^ binding with high affinity within the ATD is highly subunit selective (GluN2A ≫ GluN2B > GluN2C ≥ GluN2D; [290, 365]). The receptor is also allosterically modulated, in both positive and negative directions, by endogenous neuroactive steroids in a subunit dependent manner [153, 239]. Tonic proton blockade of NMDA receptor function is alleviated by polyamines and the inclusion of exon 5 within GluN1 subunit splice variants, whereas the non‐competitive antagonists ifenprodil and traxoprodil increase the fraction of receptors blocked by protons at ambient concentration. Inclusion of exon 5 also abolishes potentiation by polyamines and inhibition by Zn^2+^ that occurs through binding in the ATD [364]. Ifenprodil, traxoprodil, haloperidol, felbamate and Ro 8‐4304 discriminate between recombinant NMDA receptors assembled from GluN1 and either GluN2A, or GluN2B, subunits by acting as selective, non‐competitive, antagonists of heterooligomers incorporating GluN2B through a binding site at the ATD GluN1/GluN2B subunit interface [187]. LY233536 is a competitive antagonist that also displays selectivity for GluN2B over GluN2A subunit‐containing receptors. Similarly, CGP61594 is a photoaffinity label that interacts selectively with receptors incorporating GluN2B versus GluN2A, GluN2D and, to a lesser extent, GluN2C subunits. TCN 201 and TCN 213 have recently been shown to block GluN2A NMDA receptors selectively by a mechanism that involves allosteric inhibition of glycine binding to the GluN1 site [29, 101, 136, 248]. In addition to influencing the pharmacological profile of the NMDA receptor, the identity of the GluN2 subunit co‐assembled with GluN1 is an important determinant of biophysical properties that include sensitivity to block by Mg^2+^, single‐channel conductance and maximal open probablity and channel deactivation time [74, 105, 123]. Incorporation of the GluN3A subunit into tri‐heteromers containing GluN1 and GluN2 subunits is associated with decreased single‐channel conductance, reduced permeability to Ca^2+^ and decreased susceptibility to block by Mg^2+^[52, 142]. Reduced permeability to Ca^2+^ has also been observed following the inclusion of GluN3B in tri‐heteromers. The expression of GluN3A, or GluN3B, with GluN1 alone forms, in Xenopus laevis oocytes, a cation channel with unique properties that include activation by glycine (but not NMDA), lack of permeation by Ca^2+^ and resistance to blockade by Mg^2+^ and NMDA receptor antagonists [56]. The function of heteromers composed of GluN1 and GluN3A is enhanced by Zn^2+^, or glycine site antagonists, binding to the GluN1 subunit [236]. Zn^2+^ also directly activates such complexes. The co‐expression of GluN1, GluN3A and GluN3B appears to be required to form glycine‐activated receptors in mammalian cell hosts [339].

**AMPA and Kainate receptors**

All AMPA receptors are additionally activated by kainate(and domoic acid) with relatively low potency, (EC_50_^~^100 μM). Inclusion of TARPs within the receptor complex increases the potency and maximal effect of kainate [165, 260]. AMPA is weak partial agonist at GluK1 and at heteromeric assemblies of GluK1/GluK2, GluK1/GluK5 and GluK2/GluK5 [171]. Quinoxalinediones such as CNQX and NBQX show limited selectivity between AMPA and kainate receptors. Tezampanel also has kainate (GluK1) receptor activity as has GYKI53655(GluK3 and GluK2/GluK3) [171]. ATPO is a potent competitive antagonist of AMPA receptors, has a weaker antagonist action at kainate receptors comprising GluK1 subunits, but is devoid of activity at kainate receptors formed from GluK2 or GluK2/GluK5 subunits. The pharmacological activity of ATPO resides with the (S)‐enantiomer. ACET and UBP310 may block GluK3, in addition to GluK1 [13, 299]. (2S,4R)‐4‐methylglutamate (SYM2081) is equipotent in activating (and desensitising) GluK1 and GluK2 receptor isoforms and, via the induction of desensitisation at low concentrations, has been used as a functional antagonist of kainate receptors. Both (2S,4R)‐4‐methylglutamate and LY339434 have agonist activity at NMDA receptors. (2S,4R)‐4‐methylglutamate is also an inhibitor of the glutamate transporters EAAT1 and EAAT2.

**Delta subunits**

GluD1 and GluD2 comprise, on the basis of sequence homology, an ‘orphan’ class of ionotropic glutamate receptor subunit. They do not form a functional receptor when expressed solely, or in combination with other ionotropic glutamate receptor subunits, in transfected cells [403]. However, GluD2 subunits bind D‐serine and glycine and GluD2 subunits carrying the mutation A654T form a spontaneously open channel that is closed by D‐serine[272].

### Further reading on Ionotropic glutamate receptors

Filippini, A et al. (2016) The Good and the Bad of Glutamate Receptor RNA Editing. Mol Neurobiol [PMID:27766534]

Greger, IH et al. (2017) Structural and Functional Architecture of AMPA‐Type Glutamate Receptors and Their Auxiliary Proteins. Neuron 94: 713‐730 [PMID:28521126]

Hackos, DH et al. (2017) Diverse modes of NMDA receptor positive allosteric modulation: Mechanisms and consequences. Neuropharmacology 112: 34‐45 [PMID:27484578]

Huettner, JE. (2015) Glutamate receptor pores. J Physiol 593: 49‐59 [PMID:25556787]

Iacobucci, GJ et al. (2007) NMDA receptors: linking physiological output to biophysical operation. Nat Rev Neurosci 18: 236‐249 [PMID:28303017]

Krieger, J et al. (2015) Structure, Dynamics, and Allosteric Potential of Ionotropic Glutamate Receptor N‐Terminal Domains. Biophys J 109: 1136‐48 [PMID:26255587]

Mollerud, S et al. (2017) Lessons from crystal structures of kainate receptors. Neuropharmacology 112: 16‐28 [PMID:27236079]

Plested, AJ. (2016) Structural mechanisms of activation and desensitization in neurotransmitter‐gated ion channels. Nat Struct Mol Biol 23: 494‐502 [PMID:27273633]

Whitehead, G et al. (2017) Ca2+‐permeable AMPA receptor: A new perspective on amyloid‐beta mediated pathophysiology of Alzheimer's disease. Neuropharmacology 112: 221‐227 [PMID:27561971]

Yuzaki, M et al. (2017) A GluD Coming‐Of‐Age Story. Trends Neurosci 40: 138‐150 [PMID:28110935]

Zhou, HX et al. (2017) Advancing NMDA Receptor Physiology by Integrating Multiple Approaches. Trends Neurosci 40: 129‐137 [PMID:28187950]

Zhuo, M. (2017) Ionotropic glutamate receptors contribute to pain transmission and chronic pain. Neuropharmacology 112: 228‐234 [PMID:27543416]

## IP_3_ receptor

### Overview

The inositol 1,4,5‐trisphosphate receptors (IP_3_R) are ligand‐gated Ca^2+^‐release channels on intracellular Ca2+ store sites (such as the endoplasmic reticulum). They are responsible for the mobilization of intracellular Ca^2+^ stores and play an important role in intracellular Ca^2+^ signalling in a wide variety of cell types. Three different gene products (types I–III) have been isolated, which assemble as large tetrameric structures. IP_3_Rs are closely associated with certain proteins: calmodulin(CALM1 CALM2 CALM3, P62158) and FKBP (and calcineurin via FKBP). They are phosphorylated by PKA, PKC, PKG and CaMKII.

**Table:** 

Nomenclature	IP_3_R1	IP_3_R2	IP_3_R3
HGNC, UniProt	ITPR1, Q14643	ITPR2, Q14571	ITPR3, Q14573
Endogenous activators	cytosolic ATP (< mM range), cytosolic Ca^2+^ Concentration range: ¡7.5×10^−4^M, IP_3_ (endogenous; nM ‐ *μ*M range)	cytosolic Ca^2+^ (nM range), IP_3_ (endogenous; nM ‐ *μ*M range)	cytosolic Ca^2+^ (nM range), IP_3_ (endogenous; nM ‐ *μ*M range)
Activators	adenophostin A (pharmacological; nM range), inositol 2,4,5‐trisphosphate (pharmacological; also activated by other InsP_3_ analogues)	adenophostin A (pharmacological; nM range), inositol 2,4,5‐trisphosphate (pharmacological; also activated by other InsP_3_ analogues)	–
Antagonists	PIP_2_ (*μ*M range), caffeine (mM range), decavanadate (*μ*M range), xestospongin C (*μ*M range)	decavanadate (*μ*M range)	decavanadate (*μ*M range)
Functional Characteristics	Ca^2+^: (P_Ba_/P_K_^~^6) single‐channel conductance ^~^70 pS (50 mM Ca^2+^)	Ca^2+^: single‐channel conductance ^~^70 pS (50 mM Ca^2+^) ^~^390 pS (220 mM Cs^+^)	Ca^2+^: single‐channel conductance ^~^88 pS (55 mM Ba^2+^)
Comments	IP_3_ R1 is also antagonised by calmodulin at high cytosolic Ca^2+^ concentrations	–	–

### Comments

The absence of a modulator of a particular isoform of receptor indicates that the action of that modulator has not been determined, not that it is without effect.

### Further reading on IP_3_ receptor

Berridge MJ. (2016) The Inositol Trisphosphate/Calcium Signaling Pathway in Health and Disease. Physiol Rev 96: 1261‐96 [PMID:27512009]

Garcia MI et al. (2017) Cardiac inositol 1,4,5‐trisphosphate receptors. Biochim Biophys Acta 1864: 907‐914 [PMID:27884701]

Mak, DO et al. (2015) Inositol 1,4,5‐trisphosphate receptors in the endoplasmic reticulum: A single‐channel point of view. Cell Calcium 58: 67‐78 [PMID:25555684]

Prole DL et al. (2016) Inositol 1,4,5‐trisphosphate receptors and their protein partners as signalling hubs. J Physiol 594: 2849‐66 [PMID:26830355]

Seo MD et al. (2015) Structural insights into endoplasmic reticulum stored calcium regulation by inositol 1,4,5‐trisphosphate and ryanodine receptors. Biochim Biophys Acta 1853: 1980‐91 [PMID:25461839]

## Nicotinic acetylcholine receptors

### Overview

Nicotinic acetylcholine receptors are members of the Cys‐loop family of transmitter‐gated ion channels that includes the GABA_A_, strychnine‐sensitive glycine and 5‐HT_3_ receptors [6, 257, 334, 350, 387]. All nicotinic receptors are pentamers in which each of the five subunits contains four *α*‐helical transmembrane domains. Genes encoding a total of 17 subunits (*α*1‐10, *β*1‐4, *γ*, *δ* and *ϵ*) have been identified [185]. All subunits with the exception of *α*8 (present in avian species) have been identified in mammals. All *α* subunits possess two tandem cysteine residues near to the site involved in acetylcholine binding, and subunits not named *α* lack these residues [257]. The orthosteric ligand binding site is formed by residues within at least three peptide domains on the *α* subunit (principal component), and three on the adjacent subunit (complementary component). nAChRs contain several allosteric modulatory sites. One such site, for positive allosteric modulators (PAMs) and allosteric agonists, has been proposed to reside within an intrasubunit cavity between the four transmembrane domains [124, 401]; see also [144]). The high resolution crystal structure of the molluscan acetylcholine binding protein, a structural homologue of the extracellular binding domain of a nicotinic receptor pentamer, in complex with several nicotinic receptor ligands (e.g.[53]) and the crystal structure of the extracellular domain of the *α*1 subunit bound to *α*‐bungarotoxin at 1.94 Å resolution [82], has revealed the orthosteric binding site in detail (reviewed in [55, 185, 315, 334]). Nicotinic receptors at the somatic neuromuscular junction of adult animals have the stoichiometry (*α*1)_2_ *β*1*δ* *ϵ*, whereas an extrajunctional (*α*1)_2_ *β*1*γ* *δ* receptor predominates in embryonic and denervated skeletal muscle and other pathological states. Other nicotinic receptors are assembled as combinations of *α*(2‐6) and *β*(2‐4) subunits. For *α*2, *α*3, *α*4 and *β*2 and *β*4 subunits, pairwise combinations of *α* and *β* (e.g. *α*3*β*4 and *α*4*β*2) are sufficient to form a functional receptor in vitro, but far more complex isoforms may exist in vivo (reviewed in [127, 128, 257]). There is strong evidence that the pairwise assembly of some *α* and *β* subunits can occur with variable stoichiometry [e.g. (*α*4)_2_(*β*2)_2_ or (*α*4)_3_(*β*2)_2_] which influences the biophysical and pharmacological properties of the receptor [257]. *α*5 and *β*3 subunits lack function when expressed alone, or pairwise, but participate in the formation of functional hetero‐oligomeric receptors when expressed as a third subunit with another *α* and *β* pair [e.g. *α*4*α*5*α* *β*2, *α*4*α* *β*2*β*3, *α*5*α*6*β*2, see [257] for further examples]. The *α*6 subunit can form a functional receptor when co‐expressed with *β*4 in vitro, but more efficient expression ensues from incorporation of a third partner, such as *β*3 [391]. The *α*7, *α*8, and *α*9 subunits form functional homo‐oligomers, but can also combine with a second subunit to constitute a hetero‐oligomeric assembly (e.g. *α*7*β*2 and *α*9*α*10). For functional expression of the *α*10 subunit, co‐assembly with *α*9 is necessary. The latter, along with the *α*10 subunit, appears to be largely confined to cochlear and vestibular hair cells. Comprehensive listings of nicotinic receptor subunit combinations identified from recombinant expression systems, or in vivo, are given in [257]. In addition, numerous proteins interact with nicotinic ACh receptors modifying their assembly, trafficking to and from the cell surface, and activation by ACh (reviewed by [9, 183, 256]).

The nicotinic receptor Subcommittee of NC‐IUPHAR has recommended a nomenclature and classification scheme for nicotinic acetylcholine (nACh) receptors based on the subunit composition of known, naturally‐ and/or heterologously‐expressed nACh receptor subtypes [230]. Headings for this table reflect abbreviations designating nACh receptor subtypes based on the predominant *α* subunit contained in that receptor subtype. An asterisk following the indicated *α* subunit denotes that other subunits are known to, or may, assemble with the indicated *α* subunit to form the designated nACh receptor subtype(s). Where subunit stoichiometries within a specific nACh receptor subtype are known, numbers of a particular subunit larger than 1 are indicated by a subscript following the subunit (enclosed in parentheses – see also [71]).

**Table:** 

Nomenclature	nicotinic acetylcholine receptor *α*1 subunit	nicotinic acetylcholine receptor *α*2 subunit	nicotinic acetylcholine receptor *α*3 subunit	nicotinic acetylcholine receptor *α*4 subunit
HGNC, UniProt	CHRNA1, P02708	CHRNA2, Q15822	CHRNA3, P32297	CHRNA4, P43681
Commonly used antagonists	(*α*1)_2_ *β*1*γ* *δ* and (*α*1)_2_ *β*1*δ* *ϵ*: *α*‐bungarotoxin>pancuronium>vecuronium>rocuronium>tubocurarine (IC_50_ = 43 ‐ 82 nM)	*α*2*β*2: DH*β*E (K_B_ = 0.9 *μ*M), tubocurarine (K_B_ = 1.4 *μ*M); *α*2*β*4: DH*β*E (K_B_ = 3.6 *μ*M), tubocurarine (K_B_ = 4.2 *μ*M)	*α*3*β*2: DH*β*E (K_B_ = 1.6 *μ*M, IC_50_ = 2.0 *μ*M), tubocurarine (K_B_ = 2.4 *μ*M); *α*3*β*4: DH*β*E (K_B_ = 19 *μ*M, IC_50_ = 26 *μ*M), tubocurarine (K_B_ = 2.2 *μ*M)	*α*4*β*2: DH*β*E (K_B_ = 0.1 *μ*M; IC_50_ = 0.08 ‐ 0.9 *μ*M), tubocurarine (K_B_ = 3.2 *μ*M, IC_50_ = 34 *μ*M); *α*4*β*4: DH*β*E (K_B_ = 0.01 *μ*M, IC_50_ = 0.19 – 1.2 *μ*M), tubocurarine (K_B_ = 0.2 *μ*M, IC_50_ = 50 *μ*M)
Selective agonists	succinylcholine (selective for (*α*1)_2_ *β*1*γ* *δ*)	–	–	varenicline [70], rivanicline [91], TC‐2559 (*α*4*β*2) [65]
Selective antagonists	*α*‐bungarotoxin, *α*‐conotoxin GI, *α*‐conotoxin MI, pancuronium, waglerin‐1 (selective for (*α*1)_2_ *β*1*δ* *ϵ*)	–	*α*‐conotoxin AuIB (*α*3*β*4), *α*‐conotoxin MII (*α*3*β*2), *α*‐conotoxin PnIA (*α*3*β*2), *α*‐conotoxin TxIA (*α*3*β*2), *α*‐conotoxin‐GIC (*α*3*β*2)	–
Channel blockers	gallamine ((*α*1)_2_ *β*1*γ* *δ* and (*α*1)_2_ *β*1*δ* *ϵ*) (pIC_50_∼6), mecamylamine ((*α*1)_2_ *β*1*δ* *ϵ*) (pIC_50_∼5.8)	hexamethonium, mecamylamine	mecamylamine (*α*3*β*4) (pIC_50_ 6.4), mecamylamine (*α*3*β*2) (pIC_50_ 5.1), A‐867744 (*α*3*β*4) [240], NS1738 (*α*3*β*4) [361], hexamethonium (*α*3*β*4), hexamethonium (*α*3*β*2)	mecamylamine (*α*4*β*4) (pIC_50_ 5.3–6.5), mecamylamine (*α*4*β*2) (pIC_50_ 5.4–5.4), hexamethonium (*α*4*β*2) (pIC_50_ 4.5–5.2), hexamethonium (*α*4*β*4) (pIC_50_ 4), A‐867744 (*α*4*β*2) [240], NS1738 (*α*4*β*2) [361]
Allosteric modulators	–	LY2087101 (Positive) [42]	–	LY2087101 (Positive) [42]
Selective allosteric modulators	–	–	–	NS9283 (Positive) [216]
Labelled ligands	[^125^I]*α*‐bungarotoxin (Selective Antagonist), [^3^H]*α*‐bungarotoxin (Selective Antagonist)	[^125^I]epibatidine (Agonist), [^3^H]epibatidine (Agonist), [^3^H]cytisine (Agonist)	[^125^I]epibatidine (Agonist), [^3^H]epibatidine (Agonist), [^3^H]cytisine (Agonist)	[^125^I]epibatidine (Agonist), [^3^H]epibatidine (Agonist), [^3^H]cytisine (Agonist), [^125^I]epibatidine (Agonist), [^3^H]epibatidine (Agonist), [^3^H]nicotine (Agonist) – Rat, [^3^H]cytisine (Agonist)
Functional Characteristics	(*α*1)_2_ *β* *γ* *δ*: P_Ca_/P_Na_ = 0.16 ‐ 0.2, P_f_ = 2.1 – 2.9%; (*α*1)_2_ *β* *δ* *ϵ*: P_Ca_/P_Na_ = 0.65 – 1.38, P_f_ = 4.1 – 7.2%	*α*2*β*2: P_Ca_/P_Na_^~^1.5	*α*3*β*2: P_Ca_/P_Na_ = 1.5; *α*3*β*4: P_Ca_/P_Na_ = 0.78 ‐ 1.1, P_f_ = 2.7 – 4.6%	*α*4*β*2: P_Ca_/P_Na_ = 1.65, P_f_ = 2.6 – 2.9%; *α*4*β*4: P_f_ = 1.5 – 3.0 %

**Table:** 

Nomenclature	nicotinic acetylcholine receptor *α*5 subunit	nicotinic acetylcholine receptor *α*6 subunit	nicotinic acetylcholine receptor *α*7 subunit
HGNC, UniProt	CHRNA5, P30532	CHRNA6, Q15825	CHRNA7, P36544
Commonly used antagonists	–	*α*6/*α*3*β*2*β*3 chimera: DH*β*E (IC_50_ = 1.1 *μ*M)	(*α*7)_5_: DH*β*E (IC_50_ = 8 ‐ 20 *μ*M); (*α*7)_5_: tubocurarine (IC_50_ = 3.1 *μ*M)
Selective agonists	–	–	encenicline (Partial agonist) [247, 280], AQW051 ([125I]*α*‐ bungarotoxin binding assay) [167], 4BP‐TQS (allosteric) [124], A‐582941 ((*α*7)_5_) [33], PHA‐543613 ((*α*7)_5_) [385], PHA‐709829 ((*α*7)_5_) [2], PNU‐282987 ((*α*7)_5_) [36], bradanicline ((*α*7)_5_) [139]
Selective antagonists	*α*‐conotoxin MII, *α*‐conotoxin PnIA, *α*‐conotoxin TxIA, *α*‐conotoxin‐GIC	*α*‐conotoxin MII (*α*6*β*2*), *α*‐conotoxin MII [H9A, L15A] (*α*6*β*2*β*3), *α*‐conotoxin PIA (*α*6/*α*3*β*2*β*3 chimera)	*α*‐bungarotoxin ((*α*7)_5_), *α*‐conotoxin ArIB ((*α*7)_5_), *α*‐conotoxin ImI ((*α*7)_5_), methyllycaconitine ((*α*7)_5_)
Channel blockers	–	mecamylamine (*α*6/*α*3*β*2*β*3 chimera) (pIC_50_ 5), hexamethonium (*α*6/*α*3*β*2*β*3 chimera) (pIC_50_ 4)	mecamylamine ((*α*7)_5_) (pIC_50_ 4.8)
Allosteric modulators	–	–	A‐867744 (Positive) [240], LY2087101 (Positive) [42], NS1738 (Positive) [361]
Selective allosteric modulators	–	–	JNJ1930942 (Positive) [87], PNU‐120596 (Positive) [161]
Labelled ligands	–	[^3^H]epibatidine (Agonist) – Chicken, [^125^I]*α*‐conotoxin MII (Antagonist)	[^3^H]epibatidine (Agonist), [^3^H]A‐585539 (Agonist) [7], [^3^H]AZ11637326 (Agonist) [126], [^125^I]*α*‐bungarotoxin (Selective Antagonist) (p*K*_d_ 8.3–9.1), [^3^H]*α*‐bungarotoxin (Selective Antagonist) (p*K*_d_ 8.3–9.1), [^3^H]methyllycaconitine (Antagonist) (p*K*_d_ 8.7) – Rat
Functional Characteristics	–	–	P_Ca_/P_Na_ = 6.6‐20, P_f_ = 8.8 ‐ 11.4%

**Table:** 

Nomenclature	nicotinic acetylcholine receptor *α*8 subunit (avian)	nicotinic acetylcholine receptor *α*9 subunit	nicotinic acetylcholine receptor *α*10 subunit
HGNC, UniProt	–	CHRNA9, Q9UGM1	CHRNA10, Q9GZZ6
Commonly used antagonists	(*α*8)_5_: *α*‐bungarotoxin>atropine≥tubocurarine≥strychnine	(*α*9)_5_: *α*‐bungarotoxin>methyllycaconitine>strychnine ^~^tropisetron >tubocurarine; *α*9*α*10: *α*‐bungarotoxin>tropisetron = strychnine>tubocurarine	*α*9*α*10: *α*‐bungarotoxin>tropisetron = strychnine>tubocurarine
Selective antagonists	–	*α*‐bungarotoxin ((*α*9)_5_), *α*‐bungarotoxin (*α*9*α*10), *α*‐conotoxin RgIA (*α*9*α*10), muscarine ((*α*9)_5_), muscarine (*α*9*α*10), nicotine ((*α*9)_5_), nicotine (*α*9*α*10), strychnine ((*α*9)_5_), strychnine (*α*9*α*10)	*α*‐bungarotoxin (*α*9*α*10), *α*‐conotoxin RgIA (*α*9*α*10), muscarine (*α*9*α*10), nicotine (*α*9*α*10), strychnine (*α*9*α*10)
Labelled ligands	[^3^H]epibatidine ((*α*8)_5_) (p*K*_d_ 9.7), [^125^I]*α*‐bungarotoxin (native *α*8*) (p*K*_d_ 8.3), [^3^H]*α*‐bungarotoxin (native *α*8*) (p*K*_d_ 8.3)	[^3^H]methyllycaconitine (Antagonist) (p*K*_d_ 8.1), [^125^I]*α*‐bungarotoxin (Antagonist), [^3^H]*α*‐bungarotoxin (Antagonist)	[^3^H]methyllycaconitine (Antagonist) (p*K*_d_ 8.1)
Functional Characteristics	–	(*α*9)_5_: P_Ca_/P_Na_ = 9; *α*9*α*10: P_Ca_/P_Na_ = 9, P_f_ = 22%	*α*9*α*10: P_Ca_/P_Na_ = 9, P_f_ = 22%

**Table:** 

Nomenclature	nicotinic acetylcholine receptor *β*1 subunit	nicotinic acetylcholine receptor *β*2 subunit	nicotinic acetylcholine receptor *β*3 subunit	nicotinic acetylcholine receptor *β*4 subunit	nicotinic acetylcholine receptor *γ* subunit	nicotinic acetylcholine receptor *δ* subunit	nicotinic acetylcholine receptor *ϵ* subunit
HGNC, UniProt	CHRNB1, P11230	CHRNB2, P17787	CHRNB3, Q05901	CHRNB4, P30926	CHRNG, P07510	CHRND, Q07001	CHRNE, Q04844
Antagonists	–	–	–	–	–	PhTX‐11 (pIC_50_ 6.2–6.3) [346]	–
Comments	Ligand interaction data for hetero‐oligomeric receptors containing the *β*1 receptors are listed under the *α*1 receptors.						

### Comments

Commonly used agonists of nACh receptors that display limited discrimination in functional assays between receptor subtypes include A‐85380, cytisine, DMPP, epibatidine, nicotine and the natural transmitter, acetylcholine(ACh). A summary of their profile across differing receptors is provided in [128] and quantitative data across numerous assay systems are summarized in [177]. Quantitative data presented in the table for commonly used antagonists and channel blockers for human receptors studied under voltage‐clamp are from [46, 58, 291, 292, 295, 386]. Type I PAMs increase peak agonist‐evoked responses but have little, or no, effect on the rate of desensitization of *α*7 nicotinic ACh receptors whereas type II PAMs also cause a large reduction in desensitization (reviewed in [384]).

### Further reading on Nicotinic acetylcholine receptors

Auerbach, A. (2015) Agonist activation of a nicotinic acetylcholine receptor. Neuropharmacology 96: 150‐6 [PMID:25446670]

Bertrand, D et al. (2015) Therapeutic Potential of alpha7 Nicotinic Acetylcholine Receptors. Pharmacol Rev 67: 1025‐1073 [PMID:26419447]

Bouzat, C et al. (2017) Nicotinic acetylcholine receptors at the single‐channel level. Br J Pharmacol [PMID:28261794]

Chatzidaki, A et al. (2015) Allosteric modulation of nicotinic acetylcholine receptors. Biochem Pharmacol 97: 408‐17 [PMID:26231943]

Corradi, J et al. (2016) Understanding the Bases of Function and Modulation of alpha7 Nicotinic Receptors: Implications for Drug Discovery. Mol Pharmacol 90: 288‐99 [PMID:27190210]

Crespi, A et al. (2017) Proteins and chemical chaperones involved in neuronal nicotinic receptor expression and function: an update. Br J Pharmacol [PMID:28294298]

Dineley, KT et al. (2015) Nicotinic ACh receptors as therapeutic targets in CNS disorders. Trends Pharmacol Sci 36: 96‐108 [PMID:25639674]

Lukas, RJ et al. (1999) International Union of Pharmacology. XX. Current status of the nomenclature for nicotinic acetylcholine receptors and their subunits. Pharmacol Rev 51: 397‐401 [PMID:10353988]

Stokes, C et al. (2015) Looking below the surface of nicotinic acetylcholine receptors. Trends Pharmacol Sci 36: 514‐23 [PMID:26067101]

Wang, J et al. (2017) Orthosteric and allosteric potentiation of heteromeric neuronal nicotinic acetylcholine receptors. Br J Pharmacol [PMID:28199738]

Wu, J et al. (2016) Heteromeric alpha7beta2 Nicotinic Acetylcholine Receptors in the Brain. Trends Pharmacol Sci 37: 562‐74 [PMID:27179601]

## P2X receptors

### Overview

P2X receptors (**nomenclature as agreed by the NC‐IUPHAR Subcommittee on P2X Receptors** [71, 196]) have a trimeric topology [179, 191, 275] with two putative TM domains, gating primarily Na^+^, K^+^ and Ca^2+^, exceptionally Cl^‐^. The Nomenclature Subcommittee has recommended that for P2X receptors, structural criteria should be the initial criteria for nomenclature where possible. X‐ray crystallography indicates that functional P2X receptors are trimeric and three agonist molecules are required to bind to a single receptor in order to activate it [125, 138, 191, 242]. Native receptors may occur as either homotrimers (e.g. P2X1 in smooth muscle) or heterotrimers (e.g. P2X2:P2X3 in the nodose ganglion and P2X1:P2X5 in mouse cortical astrocytes, [213]. P2X2, P2X4 and P2X7 receptors have been shown to form functional homopolymers which, in turn, activate pores permeable to low molecular weight solutes [349]. The hemi‐channel pannexin‐1 has been implicated in the pore formation induced by P2X7 [298], but not P2X2 [57], receptor activation.

**Table:** 

Nomenclature	P2X1	P2X2	P2X3	P2X4	P2X5	P2X6	P2X7
HGNC, UniProt	P2RX1, P51575	P2RX2, Q9UBL9	P2RX3, P56373	P2RX4, Q99571	P2RX5, Q93086	P2RX6, O15547	P2RX7, Q99572
Endogenous agonists	–	ATP [167] – Rat	ATP [168]	ATP [168]	ATP [168] – Rat	ATP [168] – Rat	ATP [168]
Agonists	*αβ*‐meATP, BzATP, L‐*βγ*‐meATP	–	*αβ*‐meATP, BzATP	–	–	–	–
Antagonists	TNP‐ATP (pIC_50_∼8.9) [370], Ip_5_I (pIC_50_∼8.5), NF023 (pIC_50_∼6.7), NF449 (pIC_50_∼6.3) [195]	NF770 (pIC_50_ 7–8) [281], NF778 (pIC_50_ 7–8) [281], PSB‐10211 (pIC_50_∼7) [281]	TNP‐ATP (pIC_50_∼8.9) [370], AF‐906 (pIC_50_ 8.9) [170], AF‐219 (pIC_50_ 8.5) [170], A317491 (pIC_50_∼7.5) [173]	5‐BDBD (pIC_50_ 5–6) [170, 281], BX‐430 (pIC_50_ 5–6) [170, 281], PSB‐12062 (pIC_50_ 5–6) [170, 281], paroxetine (pIC_50_ 5–6) [175, 281]	–	–	AZ10606120 (p*K*_d_ 8.9) [250], A804598 (pIC_50_∼8), brilliant blue G (pIC_50_∼8) [180], A839977 (pIC_50_∼7.7) [93, 95, 149], A740003 (pIC_50_ 7.4) [150], decavanadate (pA_2_ = 7.4) (pA_2_ 7.4) [255], A438079 (pIC_50_∼6.9) [93], AZ11657312 (salt free) (pA_2_ 6.1) [11]
Selective antagonists	–	–	–	–	–	–	JNJ‐479655 (p*K*_i_ 7.9) [31]
Allosteric modulators	–	–	–	–	–	–	AZ10606120 (Negative) [250], GW791343 (Positive) [250, 252] – Rat, GW791343 (Negative) [250, 252]
Selective allosteric modulators	MRS 2219 (Positive) [169]	–	–	ivermectin (Positive) (pEC_50_∼6.6) [197] – Rat	–	–	chelerythrine (Negative) (pIC_50_ 5.2) [329], AZ11645373 (Negative) [253, 345], KN62 (Negative) [122, 329], ivermectin (Positive) [283]
Comments	–	–	–	–	–	–	Effects of the allosteric modulators at P2X7 receptors are species‐dependent.

### Comments

A317491 and RO3 also block the P2X2:P2X3 heteromultimer [113, 173]. NF449, A317491 and RO3 are more than 10‐fold selective for P2X1 and P2X3 receptors, respectively.

Agonists listed show selectivity within recombinant P2X receptors of ca. one order of magnitude. A804598, A839977, A740003 and A438079 are at least 10‐fold selective for P2X7 receptors and show similar affinity across human and rodent receptors [93, 95, 149].

Several P2X receptors (particularly P2X1 and P2X3) may be inhibited by desensitisation using stable agonists (e.g. *αβ*‐meATP); suramin and PPADS are non‐selective antagonists at rat and human P2X1–3,5 and hP2X4, but not rP2X4,6,7 [45], and can also inhibit ATPase activity [72]. Ip_5_I is inactive at rP2X2, an antagonist at rP2X3 (pIC_50_ 5.6) and enhances agonist responses at rP2X4 [199]. Antagonist potency of NF023 at recombinant P2X2, P2X3 and P2X5 is two orders of magnitude lower than that at P2X1 receptors [342]. The P2X7 receptor may be inhibited in a non‐competitive manner by the protein kinase inhibitors KN62 and chelerythrine[329], while the p38 MAP kinase inhibitor GTP*γ*S and the cyclic imide AZ11645373 show a species‐dependent non‐competitive action [94, 253, 254, 345]. The pH‐sensitive dye used in culture media, phenol red, is also reported to inhibit P2X1 and P2X3 containing channels [200]. Some recombinant P2X receptors expressed to high density bind [^35^S]ATP*γ*S and [^3^H]*αβ*‐meATP, although the latter can also bind to 5^*′*^‐nucleotidase [251]. [^3^H]A317491 and [^3^H]A804598 have been used as high affinity antagonist radioligands for P2X3 (and P2X2/3) and P2X7 receptors, respectively [95].

### Further reading on P2X receptors

Coddou C et al. (2011) Activation and regulation of purinergic P2X receptor channels. Pharmacol Rev 63: 641‐83 [PMID:21737531]

Habermacher C et al. (2016) Molecular structure and function of P2X receptors. Neuropharmacology 104: 18‐30 [PMID:26231831]

Jacobson, KA et al. (2016) Medicinal chemistry of adenosine, P2Y and P2X receptors. Neuropharmacology 104: 31‐49 [PMID:26686393]

Khakh BS et al. (2001) International Union of Pharmacology. XXIV. Current status of the nomenclature and properties of P2X receptors and their subunits Pharmacol Rev 53: 107‐118 [PMID:11171941]

North, RA. (2016) P2X receptors. Philos Trans R Soc Lond B Biol Sci 371: [PMID:27377721]

## ZAC

### Overview

The zinc‐activated channel (ZAC, **nomenclature as agreed by the NC‐IUPHAR Subcommittee for the Zinc Activated Channel**) is a member of the Cys‐loop family that includes the nicotinic ACh, 5‐HT_3_, GABA_A_ and strychnine‐sensitive glycine receptors [78, 155, 363]. The channel is likely to exist as a homopentamer of 4TM subunits that form an intrinsic cation selective channel equipermeable to Na^+^, K^+^ and Cs^+^, but impermeable to Ca^2+^ and Mg^2+^[363]. ZAC displays constitutive activity that can be blocked by tubocurarine and high concentrations of Ca^2+^[363]. Although denoted ZAC, the channel is more potently activated by protons and copper, with greater and lesser efficacy than zinc, respectively [363]. ZAC is present in the human, chimpanzee, dog, cow and opossum genomes, but is functionally absent from mouse, or rat, genomes [78, 155].

**Table:** 

Nomenclature	ZAC
HGNC, UniProt	ZACN, Q401N2
Endogenous agonists	H^+^ [363], Cu^2+^ [363], Zn^2+^ [78, 363]
Antagonists	tubocurarine (pIC_50_ 5.2) [78], Ca^2+^ (pIC_50_ 2) [363]
Functional Characteristics	Outwardly rectifying current (both constitutive and evoked by Zn^2+^)

### Comments

The ZAC subunit does not appear to exist in the mouse or rat genomes [78]. Although tabulated as an antagonist, it is possible that tubocurarine acts as a channel blocker. Antagonism by Ca^2+^ is voltage‐independent. ZAC is not activated (at 1 mM) by transition metals including Fe^2+^, Co^2+^, Ni^2+^, Cd^2+^, or Al^3+^[363]. The concentration response relationship to Cu^2+^ is biphasic, with concentrations exceeding 30 *μ*M being associated with reduced activation [363].

### Further reading on ZAC

Collingridge, GL et al. (2009) A nomenclature for ligand‐gated ion channels. Neuropharmacology 56: 2‐5 [PMID:18655795]

Peralta, FA et al. (2016) Zinc as Allosteric Ion Channel Modulator: Ionotropic Receptors as Metalloproteins. Int J Mol Sci 17: [PMID:27384555]

Trattnig, SM et al. (2016) Copper and protons directly activate the zinc‐activated channel. Biochem Pharmacol 103: 109‐17 [PMID:26872532]
